# Blockage of retinoic acid signaling via RARγ suppressed the proliferation of pancreatic cancer cells by arresting the cell cycle progression of the G1-S phase

**DOI:** 10.1186/s12935-023-02928-4

**Published:** 2023-05-17

**Authors:** Kohei Yamakawa, Michiyo Koyanagi-Aoi, Akihito Machinaga, Nobuyuki Kakiuchi, Tomonori Hirano, Yuzo Kodama, Takashi Aoi

**Affiliations:** 1 Division of Stem Cell Medicine, Graduate School of Medicine, Kobe University, 7-5-1 Kusunoki-Cho, Chuo-Ku, Kobe, Hyogo 650-0017 Japan; 2 Division of Advanced Medical Science, Graduate School of Science, Technology and Innovation, Kobe University, Kobe, Hyogo Japan; 3 Division of Gastroenterology, Department of Internal Medicine, Kobe University Graduate School of Medicine, Kobe, Hyogo Japan; 4 Center for Human Resource Development for Regenerative Medicine, Kobe University Hospital, Kobe, Hyogo Japan; 5 Oncology Tsukuba Research Department, Discovery, Medicine Creation, DHBL, Eisai Co., Ltd, Tsukuba, Ibaraki Japan; 6 Department of Pathology and Tumour Biology, Kyoto University Graduate School of Medicine, Sakyo-ku, Kyoto, Japan; 7 Department of Gastroenterology and Hepatology, Kyoto University Graduate School of Medicine, Sakyo-ku, Kyoto, Japan; 8 The Hakubi Center for Advanced Research, Kyoto University, Sakyo-ku, Kyoto, Japan

**Keywords:** Pancreatic ductal adenocarcinoma, Retinoic acid signaling, Retinoic acid receptor γ, Cell proliferation, Cell cycle

## Abstract

**Background:**

Our study and several studies have reported that in some cancers, including pancreatic ductal adenocarcinoma (PDAC), the expression of squamous lineage markers, such as esophagus-tissue-specific genes, correlated with a poor prognosis. However, the mechanism by which the acquisition of squamous lineage phenotypes leads to a poor prognosis remains unclear. We previously reported that retinoic acid signaling via retinoic acid receptor γ (RARγ signaling) determines the differentiation lineage into the esophageal squamous epithelium. These findings hypothesized that the activation of RARγ signaling contributed to acquiring squamous lineage phenotypes and malignant behavior in PDAC.

**Methods:**

This study utilized public databases and immunostaining of surgical specimens to examine RARγ expression in PDAC. We evaluated the function of RARγ signaling by inhibitors and siRNA knockdown using a PDAC cell line and patient-derived PDAC organoids. The mechanism of the tumor-suppressive effects by blocking RARγ signaling was examined by a cell cycle analysis, apoptosis assays, RNA sequencing and Western blotting.

**Results:**

RARγ expression in pancreatic intraepithelial neoplasia (PanIN) and PDAC was higher than that in the normal pancreatic duct. Its expression correlated with a poor patient prognosis in PDAC. In PDAC cell lines, blockade of RARγ signaling suppressed cell proliferation by inducing cell cycle arrest in the G1 phase without causing apoptosis. We demonstrated that blocking RARγ signaling upregulated p21 and p27 and downregulated many cell cycle genes, including cyclin-dependent kinase 2 (CDK2), CDK4 and CDK6. Furthermore, using patient-derived PDAC organoids, we confirmed the tumor-suppressive effect of RARγ inhibition and indicated the synergistic effects of RARγ inhibition with gemcitabine.

**Conclusions:**

This study clarified the function of RARγ signaling in PDAC progression and demonstrated the tumor-suppressive effect of selective blockade of RARγ signaling against PDAC. These results suggest that RARγ signaling might be a new therapeutic target for PDAC.

**Supplementary Information:**

The online version contains supplementary material available at 10.1186/s12935-023-02928-4.

## Introduction

Pancreatic ductal adenocarcinoma (PDAC) is one of the most aggressive solid tumors, with a nearly equal number of new cases and deaths each year (496,000 new cases and 466,000 deaths reported worldwide in 2020) [1], and the number of cases and deaths due to PDAC is projected to continue to increase [2]. A *KRAS*-activating mutation is found in more than 90% of PDAC cases and plays a crucial role in both the development and progression of PDAC [3, 4], and next-generation sequencing technology has revealed the accumulation of disruptions or mutations in various genes, such as *TP53*, *CDKN2A*, *SMAD4* and *RNF43*, in PDAC [4]. However, no therapeutics directly targeting products of mutant *KRAS* and other driver genes are presently available in PDAC.

In addition to genomics, several significant signaling pathways and molecules have been explored in PDAC, and a number of molecular-targeted therapies for PDAC, such as the PARP inhibitor olaparib [5] and the EGFR tyrosine kinase inhibitor erlotinib [6], have been developed. However, the efficacy of these drugs is not satisfactory. Therefore, to improve the prognosis of PDAC patients, additional potential therapeutic targets in PDAC need to be identified.

Two consensus molecular subtypes of PDAC have recently been proposed based on transcriptomic data: the “classical/progenitor type,” with a relatively favorable prognosis, and the “basal-like/squamous type,” with a poor prognosis [7]. Whereas the “classical/progenitor type” preserves the high expression of pancreatic endodermal cell-fate determinants, the “basal-like/squamous type” loses pancreatic identity and expresses many squamous lineage markers related to the upregulation of the *ΔNp63* transcription network [7]. Mutation patterns of well-known PDAC driver genes alone cannot explain the difference between these molecular subtypes of PDAC, and the determining mechanism remains incompletely understood.

We previously reported that in PDAC, increased expression of small proline-rich protein 1A, an esophagus-tissue-enriched gene not expressed in the pancreas, was associated with a poor patient prognosis [8]. We further reported that retinoic acid (RA) signaling via RA receptor γ (RARγ signaling) promoted the differentiation of human-induced pluripotent stem cells into the esophageal squamous epithelium with increasing p63 expression [9]. Based on these findings, we hypothesized that the activation of RARγ signaling was the mechanism underlying the loss of pancreatic identity, the emergence of squamous lineage phenotypes and the malignant behavior of PDAC.

RA signaling plays a role in the development and maintenance of homeostasis in various tissues [10] and exerts many biological effects, such as the induction of tumor suppressors in cancer [11]. Previous studies on PDAC attempted to activate RA signaling by adding all-trans RA (ATRA) based on data indicating attenuated signaling activity [12, 13]. Some basic research has reported the antitumor effect of ATRA treatment [14, 15], but clinical trials have not demonstrated the tumor-suppressive effects of RA on PDAC when combined with interferon-alpha or gemcitabine (Gem) [16, 17]. However, these studies have addressed neither the different functions among each RAR subtype nor the effects of suppressing RA signaling.

RA signaling is activated via three RAR subtypes (RARα, RARβ and RARγ), and RA signaling via each RAR is reported to have different functions in various tissues [10]. In PDAC cases, increased expression of RARα is reportedly associated with a better prognosis [13], suggesting that RARα is a tumor suppressor. RARβ is a well-documented tumor suppressor, and loss of RARβ expression or silencing of its regulatory regions by epigenetic mechanisms is found in many types of cancers, including PDAC [11, 18]. The antitumor function of RARβ in PDAC has also been confirmed by its overexpression *in vivo* and *in vitro* [19]. However, few studies have investigated the role of RARγ in PDAC, so the efficacy of blocking RARγ signaling in PDAC remains inconclusive.

We explored the role of RARγ signaling in the progression of PDAC. The present study determined that among RARs, RARγ was associated with a poor prognosis in PDAC patients. Our *in vitro* experiments showed that the activation of RARγ signaling was involved in the cell cycle progression of the G1-S phase in PDAC and that RARγ signaling had potential utility as a therapeutic target.

## Methods

### The Cancer Genome Atlas (TCGA) and genotype-tissue expression (GTEx) data analyses

TCGA-PAAD and GTEx datasets were downloaded from the GDC Data Portal (dbGaP accession: phs000178.v11.p8, URL: https://portal.gdc.cancer.gov/projects/TCGA-PAAD) and GTEx Portal (dbGaP accession: phs000424.v8.p2, URL: https://gtexportal.org/home/datasets), respectively. All data units were converted to transcripts per million (TPM). For prognostic analyses, TCGA-PAAD cases (pancreatic cancer patients) were classified into high- or low-expression groups based on each gene transcript level. The patients’ overall survival (OS) was compared using the Kaplan‒Meier method and log-rank test.

### Expression of tissue-specific genes in PDAC

We analyzed the protein expression coded by tissue-specific genes in PDAC. We obtained a list of tissue-specific genes with a fivefold higher fragments per kilobase of exon per million reads mapped (FPKM) level in a specific tissue than the maximal FPKM value in all other tissues based on previous reports [20, 21]. Regarding protein expression, we used data from the Human Protein Atlas (HPA) portal (https://www.proteinatlas.org/about/download). On the HPA, the protein expression determined by immunohistochemistry (IHC) was classified into four groups: “High,” “Medium,” “Low” and “not detected.” In this study, we defined genes with “High” or “Medium” protein expression in at least 1 of 8–12 PDAC sections as having “present” protein expression in PDAC and genes with “Low” or “not detected” protein expression in all 8–12 PDAC sections as having “absent” protein expression in PDAC. Tissue-specific genes without available protein expression data on HPA were excluded from our analyses.

### IHC analyses

Surgical specimens acquired from individuals with PDAC and pancreatic intraepithelial neoplasia (PanIN) who underwent pancreatectomy at Kobe University Hospital were used for IHC. The intensity of the nuclear staining was graded as 0 (negative), 1 (weak), 2 (intermediate), or 3 (strong), and the proportion of the stained cells was graded as 0 (negative), 1 (< 1%), 2 (1-10%), 3 (11-33%), 4 (34-66%) or 5 (> 66%). Calculation of the IHC score (total score) was performed by totaling the staining intensity score (0–3) and proportion (0–4), yielding a value of 0 or 2–8.

### Cell culture

We purchased human PDAC cell lines (PK-1, PK-8, KLM-1, Panc-1 and MIAPaca2) from RIKEN BioResource Research Center (RIKEN BRC, Ibaraki, Japan) and another human PDAC cell line (BxPC-3) from American Type Culture Collection (ATCC, Manassas, VA, USA). We maintained PK-1, PK-8, KLM-1, Panc-1 and BxPC-3 cells in RPMI-1640 (Nacalai Tesque, Kyoto, Japan) supplemented with 10% fetal bovine serum (FBS) (Merck KGaA, Darmstadt, Germany, or Life Technologies, Carlsbad, CA, USA), 100 U/ml penicillin (Life Technologies) and 100 µg/ml streptomycin (Life Technologies). We maintained MIAPaca2 cells in DMEM (Nacalai Tesque) supplemented with 10% FBS, 50 U/ml penicillin and 50 µg/ml streptomycin. We maintained all cell lines at 37 °C in a humidified atmosphere containing 5% CO_2_.

### Cell proliferation

Cells were seeded at 2000 cells per well in 96-well plates and treated with control (dimethyl sulfoxide [DMSO]), 2 RARγ antagonists with different structures, 20 µM LY2955303 (RARγi-1) (5984; Tocris, Bristol, UK) or 50 µM MM11253 (RARγi-2) (3822; Tocris). On days 0 (at seeding), 1 and 3, the number of viable cells was assessed by measuring cellular ATP levels using CellTiter-Glo® (Promega, Madison, WI, USA) according to the manufacturer’s instructions. The luminescence on days 1 and 3 was adjusted by the value at day 0.

For the crystal violet staining assay, cells were seeded at 2.4 × 10^4^ cells per well in 24-well plates and treated with control (DMSO), 20 µM RARγi-1 or 50 µM RARγi-2. On day 3, viable cells were stained using 0.4% crystal violet.

To assess the synergistic effects of RARγ inhibition and Gem (073-06631; FUJIFILM Wako Pure Chemical Corporation, Osaka, Japan), cells were seeded at 4000 cells per well in 96-well plates and treated with control (DMSO + phosphate-buffered saline [PBS]), 10 µM RARγi-1 + PBS, DMSO + 100 nM Gem or RARγi-1 + Gem. On day 3, the number of viable cells was assessed using CellTiter-Glo® (Promega).

### RARγ knockdown

Cells were seeded at 3 × 10^4^ cells per well in 24-well plates and then transfected the next day with 15 pmol of si-Control (Silencer Select Negative Control No. 1 siRNA 4390843; Life Technologies), si-RARγ #1 (Silencer Select s11807; Life Technologies) and #2 (Silencer Select s11808; Life Technologies) using Lipofectamine RNAiMAX Transfection Reagent (13778030; Life Technologies) according to the manufacturer’s instructions. Knockdown efficiency was verified by quantitative polymerase chain reaction (qPCR) and Western blotting at 48 and 96 h after transfection, respectively.

One day after siRNA transfection, cells under each condition were reseeded at 2500 cells per well in 96-well plates for a cell proliferation assay. On days 0 (at reseeding) and 3, the number of viable cells was assessed using CellTiter-Glo® (Promega). The luminescence on day 3 was adjusted by that at day 0.

### Cell cycle analyses and apoptosis assays

Cells were seeded at 4 × 10^5^ cells per well in 6-cm dishes and exposed to control (DMSO), 20 µM RARγi-1 or 50 µM RARγi-2 for 24 h. Cells were harvested and fixed with 70% ice-cold ethanol for 1 h for cell cycle analyses. The cells were then treated with RNase A (100 µg/ml) and stained with propidium iodide (50 µg/ml) (Dojindo Laboratories, Kumamoto, Japan). For apoptosis analyses, after seeding and drug exposure, cells were harvested and stained with propidium iodide and annexin V-FITC using a MEBCYTO® Apoptosis Kit (4700, Medical & Biological Laboratories, Tokyo, Japan) according to the manufacturer’s instructions.

The cell cycle and apoptosis status were analyzed using a FACS Verse (Becton, Dickinson and Company, Franklin Lakes, NJ, USA).

### PDAC organoids

Five patient-derived PDAC organoids (KYK070, KYK002, KYK023, KYK090 and KYK093) were cultured three-dimensionally in growth factor reduced Matrigel (354230; Corning, Corning, NY, USA) with complete organoid media containing advanced DMEM/F12 (12634-010; Life Technologies) supplemented with 10% Afamin/Wnt3a CM (J2-001; Medical & Biological Laboratories), 10% R-spondin1-conditioned medium from Cultrex R-spondin1 Cell and Reagent (3710-001-01; R&D Systems, Minneapolis, MN, USA), 10 mM HEPES (15630-080; Life Technologies), 1% GlutaMax (35050-061; Life Technologies), 2% B27 (17504044; Life Technologies), 10 nM gastrin-I (G9020-1MG; Merck KGaA), 500 mM N-acetyl-L-cysteine (017-05131; FUJIFILM Wako Pure Chemical Corporation), 10 ng/ml EGF (236-EG; R&D systems), 100 ng/ml noggin (6057-NG; R&D Systems), 1 mM A83-01 (SML0788-5MG; Merck KGaA), 100 ng/ml FGF-10 (060-04401; FUJIFILM Wako Pure Chemical Corporation) and 10 mM nicotinamide (N0636; Merck KGaA). Media were replaced every two to three days. All organoids were maintained at 37 °C in a humidified atmosphere containing 5% CO_2_.

### Organoid assays

1. To assess the effects of RARγ inhibition, 2500 organoid cells were cultured in 25 µl Matrigel (354230; Corning) and treated with control (DMSO), 20 µM RARγi-1 or 50 µM RARγi-2 for 10 days.
2. To assess the synergistic effects of RARγ inhibition and Gem, 2500 organoid cells were cultured in 25 µl Matrigel (354230, Corning) and treated with control (DMSO + PBS), 10 µM RARγi-1 + PBS, DMSO + 4 nM Gem or RARγi-1 + Gem for 10 days. On day 10, the number of viable cells was determined using CellTiter-Glo® (Promega).
3. To assess the effects of RARγ inhibition on cell cycle progression, KYK070 organoids were treated with control (DMSO), RARγi-1 or RARγi-2 after forming their lumen, and Ki67 and pan-cytokeratin AE1/AE3 staining was performed 24 h after drug exposure.

The numbers of Ki67^+^ cells and AE1/AE3^+^ cells forming the lumen of the organoid gland duct (referred to as “total cells”) were manually counted in each glandular lumen of the organoids, and the Ki67^+^ cell ratio was calculated using the following equation: Ki67^+^ cell ratio = the number of Ki67^+^ cells/total cells.

### Statistical analyses

Statistical analyses were performed using the GraphPad Prism 8 software program (GraphPad Software, La Jolla, CA, USA). The results are shown as the mean ± standard deviation (SD) of three or four independent experiments. Two-tailed *t* tests were used for the statistical comparison between two groups, and an analysis of variance (ANOVA) followed by Tukey’s or Dunnett’s multiple-comparison test was used for statistical comparisons between more than two groups. For the pairs for which Tukey’s or Dunnett’s multiple-comparison test indicated no significant difference, the notations “not significant” were omitted from the figures. Kaplan–Meier estimates were compared with the log-rank test. A *P* value less than 0.05 (*p* < 0.05) was considered statistically significant.

### Additional methods

Genetic information on four major driver genes of PK-1, Panc-1 and PDAC organoids is listed in Table S1 [22, 23]. Additional methods, including those for IHC, reverse transcription (RT)-PCR, Western blotting and RNA sequencing (RNA-seq), have been reported previously [8, 24]. For RT‒PCR, IHC and Western blotting, the PCR primers used as well as the primary and secondary antibodies are listed in Tables S2, S3 and S4. The visualized signals were quantified using ImageJ software (National Institutes of Health, Bethesda, MD, USA) [25]. For RNA-seq analyses, wikipathway and gene-set enrichment analysis (GSEA) of the obtained data were performed using the Strand NGS software program (Strand Life Science) and the GSEA software program (a joint project of UC San Diego and Broad Institute) [26], respectively. The RNA-seq data have been registered in Gene Expression Omnibus (GEO) at GSE210112.

## Results

### Overexpression of RARγ and esophagus-tissue-specific genes in pancreatic cancer is associated with a poor patient prognosis

**Fig. 1:**
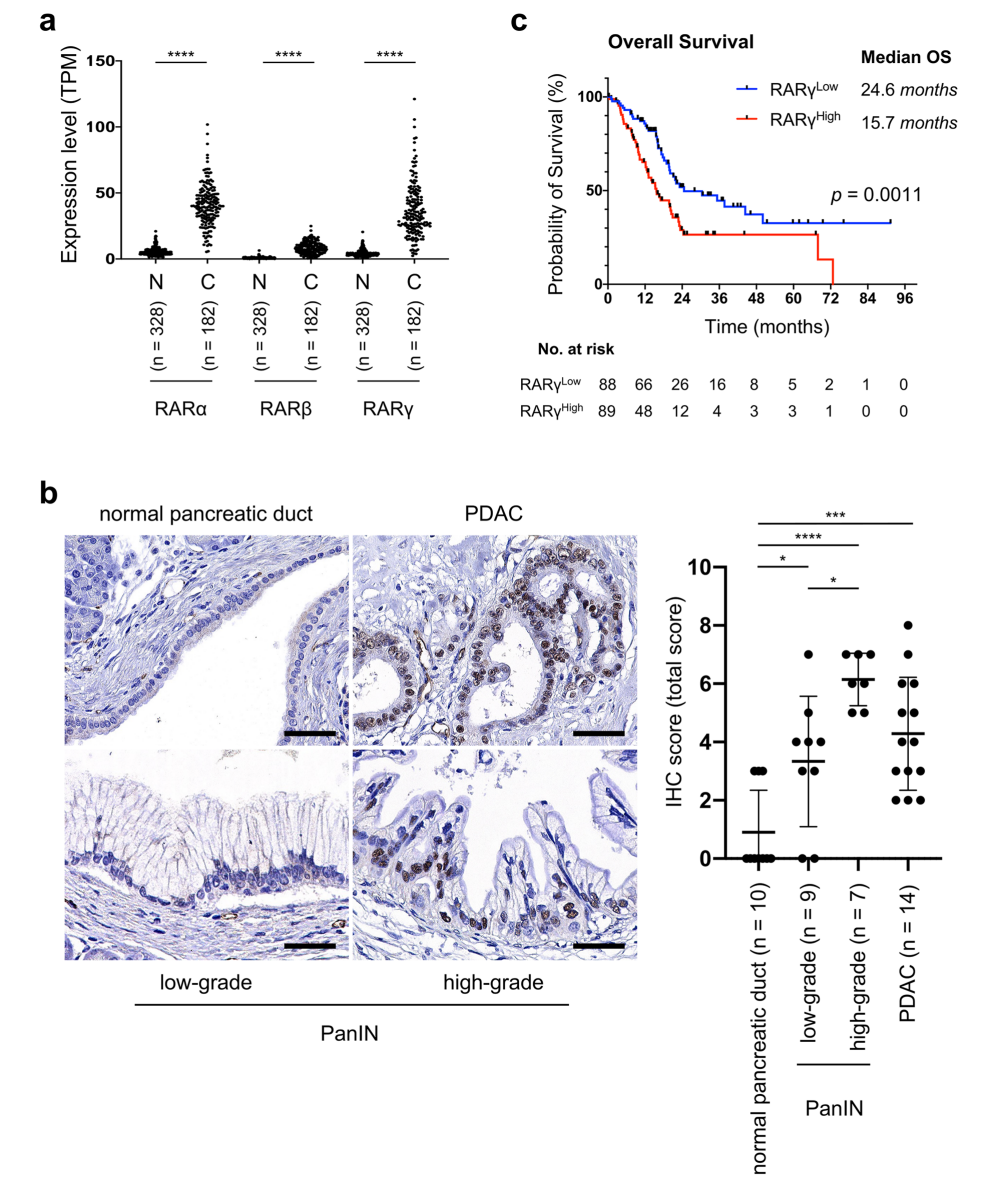
RARγ was overexpressed in PDAC and correlated with a poor prognosis. **a** The transcript levels of RARs between normal pancreatic tissues (N) and pancreatic cancers (C) were compared by analyzing TCGA-PAAD and GTEx data. **b** Left, representative images of RARγ IHC staining in PDAC at different stages of cancer progression. Right, IHC score for RARγ in normal pancreatic ducts, low-grade and high-grade PanINs and PDAC. **c** Pancreatic cancer patients were classified into RARγ^High^ or RARγ^Low^ groups based on the transcript levels of RARγ in TCGA-PAAD data, followed by estimation of patient OS using Kaplan‒Meier survival analysis. Scale bars, 100 μm; magnification, × 400. Error bars in **b**, mean ± SD; *p < 0.05, **p < 0.01, ***p < 0.001, ****p < 0.0001; unpaired t test (N vs. C) in **a**, one-way ANOVA with Tukey’s test in **b**, or log-rank test in **c.**

First, to explore the expression of RARs in pancreatic cancer, we compared the transcript levels of RARs between normal pancreas tissues (N) and pancreatic cancers (C) by analyzing two databases: TCGA and GTEx. Our database analyses indicated that the transcript levels of RARα and RARγ in pancreatic cancer (C) were significantly higher than those in normal pancreatic tissue (N) (RARα, mean TPM 41.1 vs. 5.6, p < 0.0001; RARγ, mean TPM 36.6 vs. 4.2, p < 0.0001) and that the transcript level of RARβ was low in both pancreatic cancer (C) and normal pancreatic tissue (N) (mean TPM 8.5 vs. 0.7, p < 0.0001) (Fig. 1a). We further examined the protein expression of RARγ in PDAC, PanIN (precancerous lesion of PDAC) and adjacent normal pancreatic ductal epithelium by IHC staining of surgical specimens (Fig. 1b). IHC results demonstrated that the IHC scores of RARγ were significantly higher in PDACs, high-grade PanINs and low-grade PanINs than in normal pancreatic ductal epithelium (normal pancreatic ductal epithelium vs. low-grade PanIN, p < 0.05; normal pancreatic ductal epithelium vs. high-grade PanIN, p < 0.0001; normal pancreatic ductal epithelium vs. PDAC, p < 0.001) (Fig. 1b).

We next analyzed TCGA-PAAD data to explore the association between the expression of RARs and patient prognosis. In TCGA analyses, the expression of RARα and RARβ did not correlate with the prognosis of pancreatic cancer patients (S-Fig. 1a, b). However, the high RARγ-expression group had a significantly worse prognosis than the low-expression group in pancreatic cancer (median OS 15.7 vs. 24.6 months, p = 0.0011) (Fig. 1c). These results suggested that the activation of RARγ signaling might contribute to PDAC progression.

**Fig. 2:**
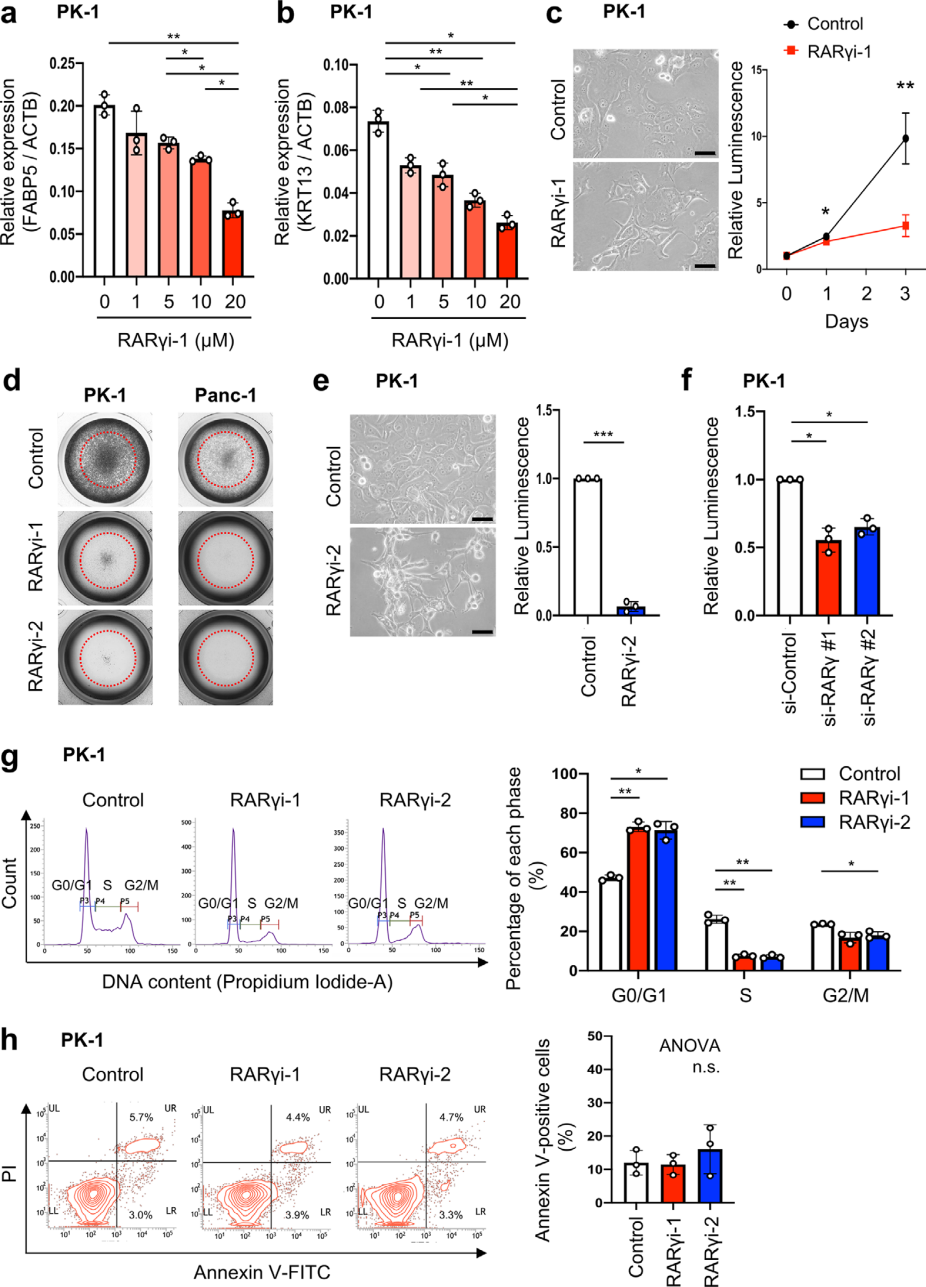
Blockage of RARγ signaling suppressed cell proliferation by inducing G1 arrest without causing cancer cell death in PDAC. **a, b** The transcript levels of FABP5 (**a**) and KRT13 (**b**), normalized to ACTB, were measured using qPCR after PK-1 cells were treated with RARγi-1 for 24 h. **c** Left, representative images on day 3 after RARγi-1 treatment. Right, the number of viable cells was assessed on days 0, 1 and 3 after RARγi-1 treatment by an ATP assay, and the luminescence on day 0 was normalized to 1. **d** Representative images of crystal violet staining on day 3 after RARγ inhibition in PK-1 and Panc-1 cells. **e** Left, representative images on day 3 after RARγi-2 treatment. Right, the number of viable cells was assessed on day 3 after RARγi-2 treatment by an ATP assay. **f** The effect of RARγ knockdown on cell proliferation was assessed by an ATP assay. **g** Left, representative images of a cell cycle analysis 24 h after RARγ inhibition in PK-1 cells. Right, the percentages of cells from each phase of the cell cycle 24 h after RARγ inhibition are shown from three independent experiments. **h** Left, representative images of a flow cytometry analysis 24 h after RARγ inhibition in PK-1 cells. Right, the percentages of annexin V-positive PK-1 cells 24 h after RARγ inhibition are shown from three independent experiments. Scale bars, 50 μm in **c, e**. Error bars in **a-c, e-h**, mean ± SD of three independent experiments; *p < 0.05, **p < 0.01, ***p < 0.001, ****p < 0.0001; n.s., not significant; by one-way ANOVA with Tukey’s test in **a, b**, paired t test in **c, e**, one-way ANOVA with Dunnett’s test (compared to control) in **f, g**, or one-way ANOVA in **h.**

We previously reported that RARγ signaling determines the differentiation lineage into the esophageal epithelium [9]. To reveal whether the expression of esophagus-tissue-specific genes is elevated in PDAC through the activation of RARγ signaling, we investigated tissue-specific genes whose protein was expressed in PDAC using the HPA. Our analysis revealed that PDAC expressed many esophagus-tissue-specific genes (12 of 28 genes) as well as other tissue-specific genes (7 of 15 adipose tissue-specific genes, 5 of 32 adrenal-specific genes, 4 of 5 gallbladder-specific genes, 3 of 24 stomach-specific genes, 2 of 13 lung-specific genes, 1 of 6 duodenum-specific protein, and 0 of 1 small intestine-specific gene) (S-Fig. 1c and Table S5). We further explored whether the expression of esophagus-tissue-specific genes was associated with patient prognosis, similar to RARγ. TCGA analyses showed that among 12 esophagus-tissue-specific genes expressed by PDAC, the increased expression of 7 significantly correlated with a poor prognosis of PDAC patients (ECM1; median OS 17.3 vs. 23.0 months, p = 0.0141, KRT13; median OS 15.8 vs. 37.7 months, p < 0.0001, KRT6A; median OS 16.2 vs. 23.4 months, p = 0.0141, ERO1L; median OS 17.7 vs. 23.2 months, p = 0.0177, FGFBP1; median OS 17.7 vs. 23.4 months, p = 0.0052, PADI1; median OS 17.3 vs. 30.4 months, p = 0.0022, GJB2; median OS 16.6 vs. 35.3 months, p = 0.0002; all data are listed in order of high-expression group vs. low-expression group) (S-Fig. 2a-l). These results might indirectly support our hypothesis that the activation of RARγ signaling drives the progression of PDAC.

### Blockage of RARγ signaling suppressed the proliferation of PDAC cells

We designed *in vitro* experiments using PDAC cell lines to elucidate the function of RARγ signaling in PDAC.

**Fig. 3:**
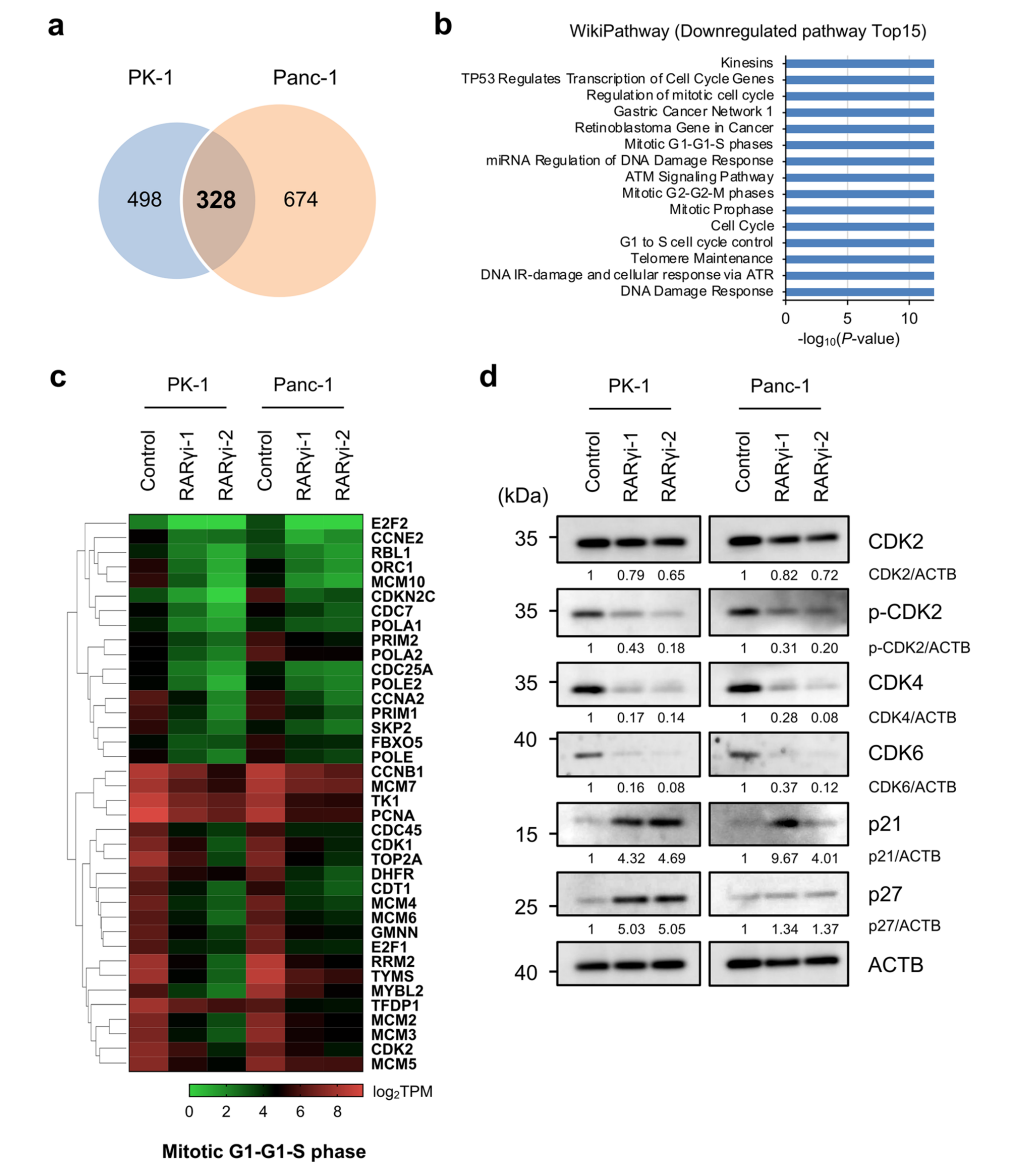
Blockage of RARγ signaling broadly downregulated the gene expression associated with the cell cycle progression of the G1-S phase and DNA synthesis in PDAC cells. **a** Venn diagrams show the entities whose expression was downregulated by RARγ inhibition in PK-1 and Panc-1 cells. **b** The blue bars indicate the top 15 pathways downregulated by RARγ inhibition. **c** The heatmap of the RNA sequencing experiment shows the expression of entities matched to Mitotic G1-G1-S phases (WP1858) in **b**. **d** The expression and phosphorylation of proteins related to G1-S phase progression were assessed using Western blotting 24 h after RARγ inhibition in PK-1 and Panc-1 cells.

First, to check whether the RARγ antagonist LY2955303 (RARγi-1) could block RARγ signaling in a PDAC cell line (PK-1), we examined the expression of FABP5, a known target gene of RA signaling [9], and the expression of KRT13, which is considered to lie downstream of RARγ [9, 27], using qPCR. RARγi-1 decreased the transcript and protein levels of both FABP5 and KRT13 (Fig. 2a, b, S-Fig. 3a, b). These findings indicated that RARγi-1 blocked RARγ signaling.

Next, to clarify the function of RARγ signaling in PDAC, we evaluated the effect of RARγ inhibition on the proliferation of a PDAC cell line (PK-1) *in vitro*. Cell proliferation assays revealed that the proliferation of PDAC cells significantly decreased in the presence of RARγi-1 compared to its absence at 24 and 72 h after seeding (p < 0.05 at 24 h, n = 3; p < 0.01 at 72 h, n = 3) (Fig. 2c, d, S-Fig. 3c). In addition, the proliferation suppressive effect was dependent on the concentration of RARγi-1 (S-Fig. 3d). To exclude the possibility that some off-target effect of RARγi-1 contributed to the results of the experiments, we re-evaluated the effect of RARγ inhibition using another RARγ antagonist, MM11253 (RARγi-2), and knockdown of RARγ. RARγi-2 suppressed the proliferation of PDAC cells (p < 0.001, n = 3) (Fig. 2d, e, S-Fig. 3c), and its effect depended on the concentration of RARγi-2, similar to RARγi-1 (S-Fig. 3e). We used small interfering RNA (siRNA) to knock down RARγ. Transfection with si-RARγ #1 or #2 decreased the mRNA and protein expression of RARγ (S-Fig. 3f, g). RARγ knockdown (si-RARγ #1 or #2) also significantly suppressed the proliferation of PDAC cells (si-Control vs. si-RARγ #1, p < 0.05, n = 3; si-Control vs. si-RARγ #2, p < 0.05, n = 3) (Fig. 2f).

To confirm whether RARγ inhibition affects other PDAC cells, we examined the expression of RARγ in various pancreatic cancer cell lines. Some lines (Panc-1, MIAPaca2 and BxPC-3) had high expression of RARγ as well as PK-1, and other lines (PK-8 and KLM-1) had low expression of RARγ (S-Fig. 3h, i). We used another line with high RARγ expression (Panc-1) and carried out the same experiments as described above. The proliferation of Panc-1 cells significantly decreased in the presence of either RARγi-1 or RARγi-2 compared to that in their absence at 72 h after seeding (Panc-1: RARγi-1 vs. control p < 0.01, RARγi-2 vs. control, p < 0.01, n = 3) (Fig. 2d, S-Fig. 3j). Our data indicated that the activation of RARγ signaling was involved in proliferation in PDAC.

### Blockage of RARγ signaling induced cell cycle arrest in the G1 phase without causing cell death in PDAC cells

**Fig. 4:**
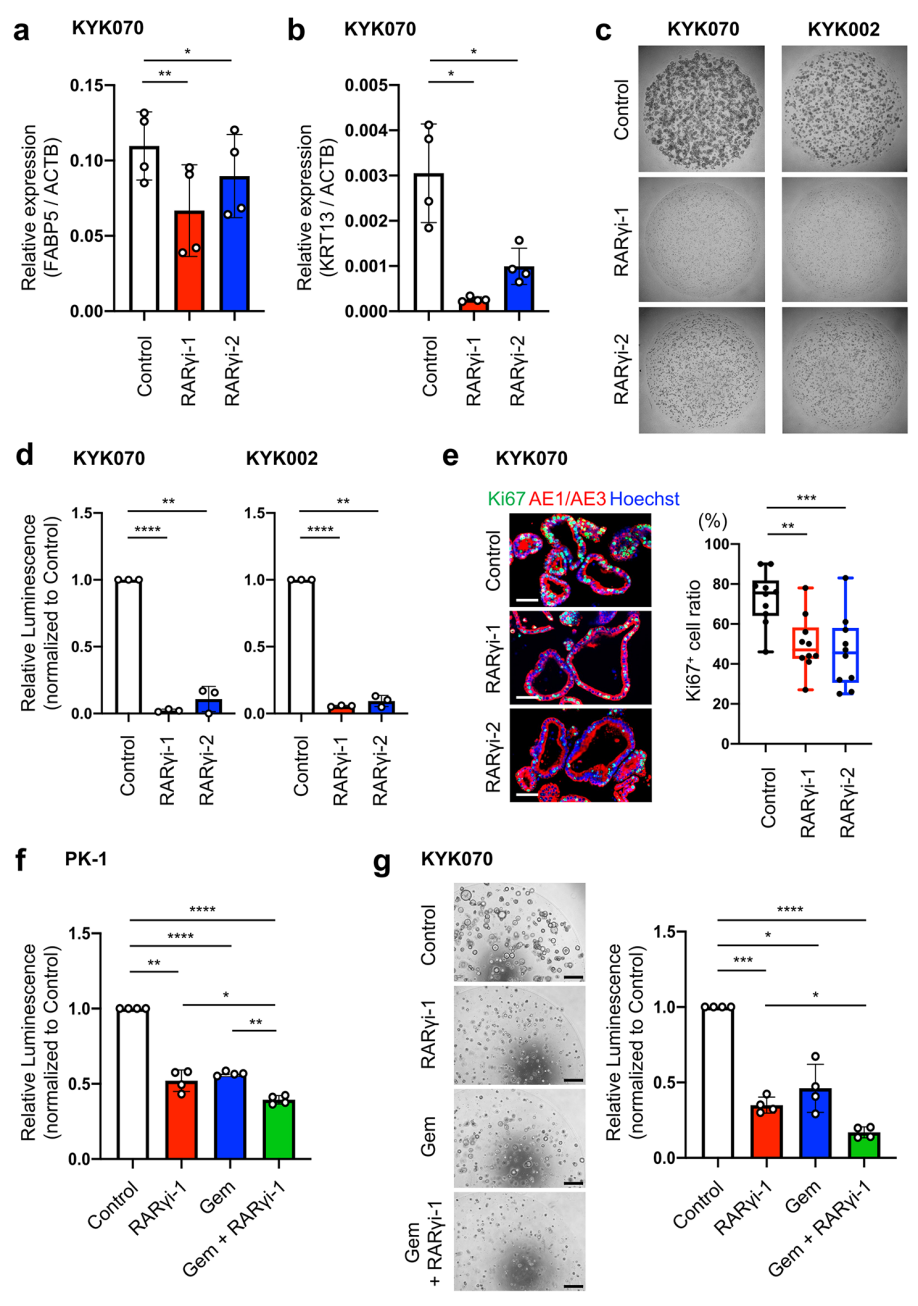
Blockage of RARγ signaling suppressed the proliferation of patient-derived PDAC organoids and synergized with Gem to inhibit cancer cell proliferation. **a, b** The transcript levels of FABP5 (**a**) and KRT13 (**b**), normalized to ACTB, were measured using qPCR 24 h after RARγ inhibition in KYK070 organoids. **c** Representative images after KYK070 and KYK002 organoids were treated with RARγi-1 or RARγi-2 for 10 days. **d** The number of viable cells was assessed by an ATP assay after RARγ inhibition for 10 days in KYK070 and KYK002 organoids. **e** Left, representative images of IHC. Right, KYK070 organoids were treated with RARγi-1 or RARγi-2 after forming their lumen, and Ki67 and pan-cytokeratin AE1/AE3 staining was performed 24 h after RARγi treatment. The Ki67^+^ cell ratio was calculated using the following equation: Ki67^+^ cell ratio = the number of Ki67^+^ cells/total cells. **f** PK-1 cells were treated with vehicle or 100 nM Gem with/without 10 µM RARγi-1 for 3 days, and then the number of viable cells was assessed by an ATP assay. **g** Left, representative images after Gem + 10 µM RARγi-1 treatment for 10 days. Right, KYK070 organoids were treated with vehicle or 4 nM Gem with/without 10 µM RARγi-1 for 10 days, and then the number of viable cells was assessed by an ATP assay. Scale bars, 50 μm in **e** and 500 μm in **g**. Error bars in **a, b, d-g**, mean ± SD of three or four independent experiments; *p < 0.05, **p < 0.01, ***p < 0.001, ****p < 0.0001; by one-way ANOVA with Dunnett’s test (compared to control) in **a, b, d, e**, or one-way ANOVA with Tukey’s test in **f, g.**

To identify the mechanism underlying growth suppression by RARγ inhibition in PDAC cells, we performed a cell cycle analysis. In PK-1 cells, RARγ inhibition by either RARγi-1 or RARγi-2 induced an increase in G0/G1-phase cells (RARγi-1-treated 73.1% vs. control 47.2%, p < 0.01, n = 3; RARγi-2-treated 71.3% vs. control 47.2%, p < 0.05, n = 3) and a decrease in S-phase cells (RARγi-1-treated 7.5% vs. control 26.2%, p < 0.01, n = 3, RARγi-2-treated 7.1% vs. control 26.2%, p < 0.01, n = 3) (Fig. 2g). In Panc-1 cells, RARγ inhibition by either RARγi-1 or RARγi-2 induced a similar result to PK-1 (G0/1-phase cells: RARγi-1-treated 69.2% vs. control 44.3%, p < 0.05, n = 3; RARγi-2-treated 62.0% vs. control 44.3%, not significant, n = 3; S-phase cells: RARγi-1-treated 5.5% vs. control 22.9%, p < 0.01, n = 3; RARγi-2-treated 9.3% vs. control 22.9%, p < 0.01, n = 3) (S-Fig. 4a). These results indicated that the blockade of RARγ signaling induced cell cycle arrest in the G1 phase in PDAC cells.

To determine whether the antineoplastic effect of RARγ inhibition was mediated by apoptosis, we performed annexin V staining. In PK-1 cells, the percentage of annexin V-positive cells among cells treated with either RARγi-1 or RARγi-2 was not higher than that among untreated cells (control 12.0% vs. RARγi-1-treated 11.5% vs. RARγi-2-treated 16.1%, not significant, n = 3) (Fig. 2h). In Panc-1 cells, RARγ inhibition by either RARγi-1 or RARγi-2 did not significantly increase annexin V-positive cells, similar to PK-1 (control 13.0% vs. RARγi-1-treated 11.9% vs. RARγi-2-treated 21.9%, not significant, n = 3) (S-Fig. 4b). This result indicated that blockade of RARγ signaling did not induce apoptosis in PDAC.

### RARγ signaling did not cross-talk with the mitogen-activated protein kinase (MAPK) pathway

The RAS-RAF-MEK-MAPK signaling pathway is a core signaling pathway that is genetically altered in most PDAC and is strongly involved in proliferation in PDAC [3, 28]. A previous study reported that the inhibition of MEK, an essential effector of the MAPK pathway [29], caused cell cycle arrest in the G1 phase, similar to our findings concerning RARγ inhibition [30].

**Fig. 5:**
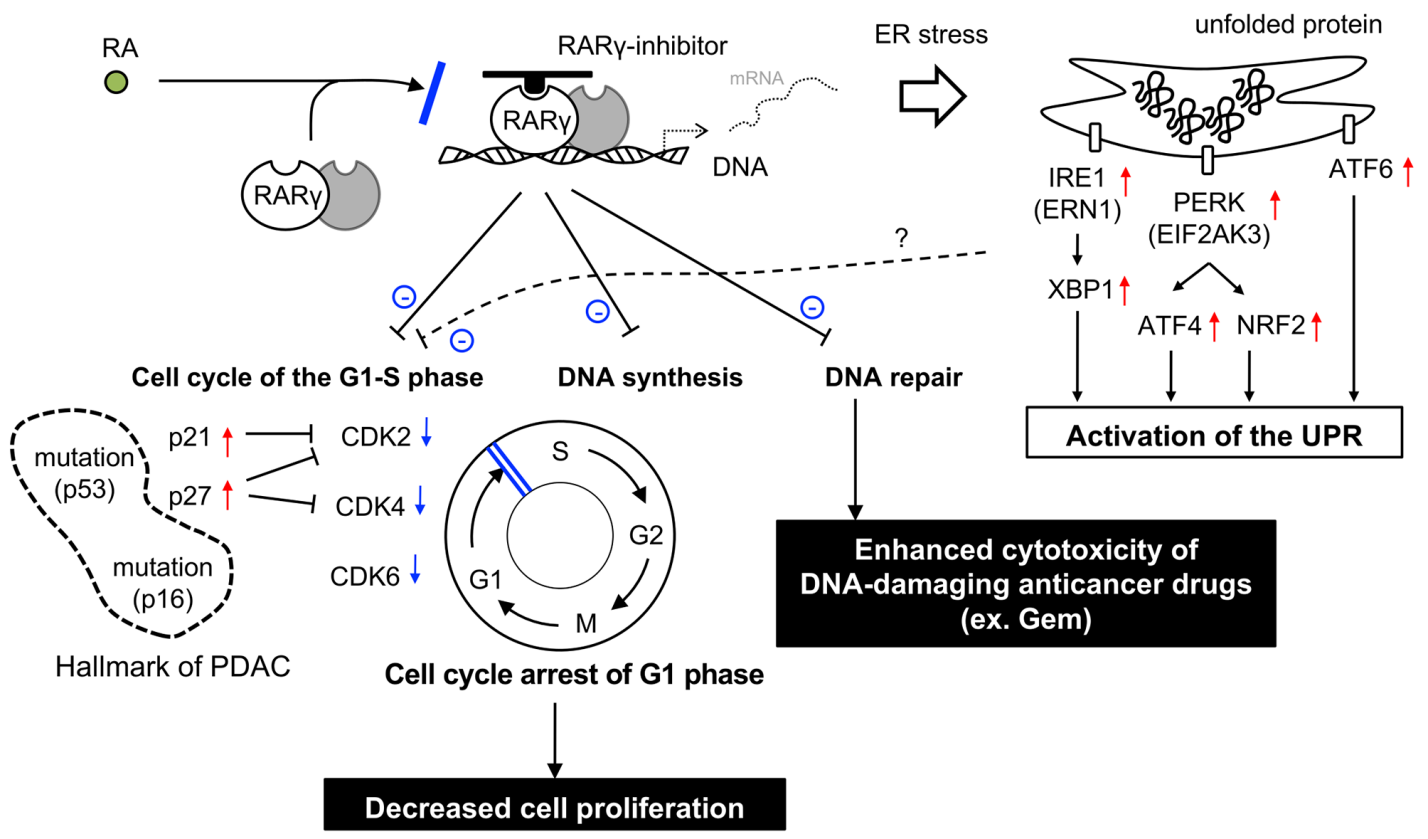
Schematic illustration of how RARγ signaling affects PDAC in the presence of a RARγ inhibitor

To clarify whether RARγ signaling cross-talks with the MAPK pathway, we examined the changes in the expression and phosphorylation of ERK1 and ERK2 (ERK1/2), which are signal-regulated kinases activated through the MAPK pathway [29], by RARγ inhibition using Western blotting in PDAC cells (PK-1 and Panc-1) (S-Fig. 5a). The expression and phosphorylation of ERK1/2 in cells treated with either RARγi-1 or RARγi-2 were not significantly decreased compared to untreated cells (PK-1, not significant, n = 3; Panc-1, not significant, n = 3) (S-Fig. 5b-e). These results suggested that RARγ signaling is involved in the proliferation of PDACs independently of the MAPK pathway.

### Blockage of RARγ signaling broadly downregulated the gene expression associated with the cell cycle progression of the G1-S phase and DNA synthesis in PDAC cells

To further understand the molecular mechanism by which RARγ inhibition affects cell proliferation, including the cell cycle process, we carried out RNA-seq using RARγi-1- or RARγi-2-treated cells (PK-1 and Panc-1).

First, we compared the gene expression between the control and RARγi-1-treated cells or between the control and RARγi-2-treated cells in each cell line. In PK-1 and Panc-1 cells, we identified 826 and 1002 entities, respectively, that were commonly downregulated more than 2-fold in RARγi-1- and RARγi-2-treated cells (S-Fig. 6a). Next, to narrow down the specific genes and pathways associated with RARγ signaling, we focused on 328 entities that were downregulated in 2 cell lines (PK-1 and Panc-1) (Fig. 3a) and performed a pathway analysis. A WikiPathway analysis revealed that RARγ inhibition significantly downregulated many pathways related to the cell cycle and DNA repair, such as G1 to S cell cycle control (WP45, p < 1.00E-12), Cell Cycle (WP179, p < 1.00E-12), Mitotic G1-G1-S phases (WP1858, p < 1.00E-12), Regulation of DNA Replication (WP1898, p < 1.00E-12) and DNA Damage Response (WP707, p < 1.00E-12) (Fig. 3b and Table S6). A further query of genes included in these pathways indicated that the gene expression associated with the cell cycle progression of the G1-S phase and DNA synthesis was broadly downregulated (Fig. 3c, Mitotic G1-G1-S phases [WP1858]). In addition, GSEA also confirmed that gene sets related to the cell cycle or DNA replication were downregulated in RARγi-1- and RARγi-2-treated cells (S-Fig. 6b, c). These findings were consistent with our experimental results that the blockade of RARγ signaling caused cell cycle arrest in the G1 phase.

As in the method described above, we also investigated the genes upregulated by RARγ inhibition. We focused on 460 commonly upregulated entities in 2 RARγi-1- and RARγi-2-treated cell lines (PK-1 and Panc-1) (S-Fig. 7a, b) and performed a pathway analysis. A WikiPathway analysis revealed that RARγ inhibition significantly upregulated many pathways related to the unfolded protein response (UPR), such as ATF4 activates genes (WP2753, p < 1.00E-12), XBP1(S) activates chaperone genes (WP3472, p < 1.00E-12), the NRF2 pathway (WP2884, p < 1.00E-12) and ATF6 (ATF6-alpha) activates chaperone genes (WP2655, p = 8.37E-08) (S-Fig. 7c, d). These findings indicated that blockade of RARγ signaling activated UPR pathways in PDAC cells, suggesting that the activation of the UPR might also partially contribute to the tumor-suppressive effects of RARγ inhibition, as ER stress and subsequent activation of the UPR are reported to cause G1 arrest [31].

Next, we examined the expression and phosphorylation of proteins associated with the cell cycle progression of the G1-S phase using Western blotting. We identified an increase in the expression of endogenous cyclin-dependent kinase (CDK) inhibitor p21, a tendency of increase in p27 and a significant decrease in the phosphorylation of CDK2 and the expression of CDK4 and CDK6 (Fig. 3d, S-Fig. 8a-g). These findings also confirmed our experimental results.

Furthermore, to explore whether RARγ inhibition affected the expression of esophagus-tissue-specific genes in PDAC cells, we first investigated the correlation between RARγ and esophagus-tissue-specific genes using TCGA-PAAD data. Our analysis showed a positive correlation between RARγ and almost all esophagus-tissue-specific genes (S-Fig. 9a). Next, we created a heatmap of the expression of esophagus-tissue-specific genes using RNA-seq data. A heatmap analysis showed that RARγ inhibition reduced the expression of only a few esophagus-tissue-specific genes (S-Fig. 9b, c). These results suggested that RARγ signaling is not the only regulator of the expression of esophagus-tissue-specific genes in PDAC.

### RARγ signaling underlies the proliferation of patient-derived PDAC organoids

To investigate whether the findings obtained from PDAC cell lines could be applied to patient-derived PDAC, we tested the effect of RARγ inhibition using established patient-derived PDAC organoids (KYK070, KYK002, KYK023, KYK090 and KYK093).

First, to clarify whether RARγ antagonists could block RARγ signaling in PDAC organoids, we examined the expression of FABP5 and KRT13 in KYK070 and KYK002 using qPCR. Both RARγi-1 and RARγi-2 decreased the transcript levels of FABP5 and KRT13 (KYK070; Fig. 4a, b and KYK002; S-Fig. 10a, b). These findings indicated that RARγ antagonists blocked RARγ signaling in PDAC organoids. Next, we evaluated the effect of RARγ inhibition on the proliferation of PDAC organoids. Cell proliferation assays revealed that the proliferation of all PDAC organoids (KYK070, KYK002, KYK023, KYK090 and KYK093) was significantly decreased in the presence of RARγi-1 or RARγi-2 compared to their absence at 10 days after seeding (Fig. 4c, d, S-Fig. 10c). Furthermore, treating lumen-forming KYK070 organoids with RARγi-1 or RARγi-2 significantly reduced the Ki67^+^ cell ratio without disrupting lumen formation (Fig. 4e). These results supported the notion that activation of RARγ signaling was involved in proliferation in patient-derived PDAC.

### Blockage of RARγ signaling synergized with chemotherapy to suppress the proliferation of PDAC cells and patient-derived PDAC organoids

To investigate whether RARγ inhibition synergizes with Gem, a critical anticancer drug for PDAC chemotherapy [32], we tested the combined effect of RARγi-1 and Gem on PDAC cells (PK-1) and patient-derived PDAC organoids (KYK070).

In PK-1 cells, RARγi-1 and Gem alone inhibited cell proliferation, and their combination significantly enhanced the suppressive effects (RARγi-1 vs. Gem + RARγi-1, p < 0.05; Gem vs. Gem + RARγi-1, p < 0.01) (Fig. 4f). Next, we confirmed the suppressive effect of RARγi-1 on the proliferation of KYK070 and decided to use 10 µM RARγi-1 for the assay (S-Fig. 10d). In KYK070, the combined suppressive effect of RARγi-1 and Gem was also observed compared to RARγi-1 alone, although there was no significant difference between the effects of Gem and Gem + RARγi-1 (RARγi-1 vs. Gem + RARγi-1, p < 0.05; Gem vs. Gem + RARγi-1, not significant) (Fig. 4g). These findings suggested that blockade of RARγ signaling might substantially impact PDAC therapy.

## Discussion

In the current study, we demonstrated that RARγ expression increased upon transformation from normal pancreatic ductal epithelium to low-grade PanIN, high-grade PanIN and PDAC using human PDAC specimens. Furthermore, the increased expression of RARγ in PDAC correlated with a worse patient prognosis. RARγ has been previously reported as an oncogene in several cancers, including cholangiocarcinoma [33], hepatocellular carcinoma [34] and colorectal cancer [35]; however, in those cancers, the nuclear receptor RARγ was overexpressed in the cytoplasm rather than in the nucleus for unknown reasons. These findings may imply not the activation of RARγ signaling but rather the abnormal transportation of RARγ into the nucleus. In October 2022, another research group reported that RARγ plays a pivotal role in the proliferation of PDAC cell lines [36]. However, the group has not performed experiments using human samples, including human PDAC specimens. Thus, to our knowledge, the current study is the first to demonstrate—in human PDAC specimens—that RARγ was overexpressed in the nucleus of cancer cells and that the expression of RARγ increased during the progression of PDAC, including PanIN, a precancerous lesion of PDAC.

Our *in vitro* study revealed that selective blockade of RARγ signaling suppressed the proliferation of PDAC cells. In addition, our database analyses showed that the expression of RARα was increased in pancreatic cancer, similar to that of RARγ, and that patient prognosis correlated with RARγ expression but not with RARα expression. Similar to PDAC cells, prostate cancer cells also express both RARα and RARγ, but the proliferation of tumor cells has been reported to depend only on RARγ [37]. The authors mentioned that these were because RARα, which requires approximately 100-times higher levels of ATRA than RARγ, was not sufficiently activated due to low levels of ATRA in prostate cancer tissues [37]. Previous studies have shown that PDAC tissues also have low levels of ATRA [13] and attenuated RA signaling activity [12]. Low ATRA levels in PDAC tissues might also be sufficient for activating RARγ but insufficient for activating RARα. We further noted that the activation of RARα signaling suppressed cell proliferation in a PDAC cell line (data not shown). Based on our findings and previously reported findings, we hypothesized that both the activation of RARγ signaling and the suppression of RARα signaling might have significance for PDAC cells. However, further studies are needed to validate our hypothesis and comprehensively understand the role of RA signaling in PDAC.

Regarding the molecular mechanism by which the blockade of RARγ signaling suppressed cell proliferation, we suggested that RARγ signaling was involved in the cell cycle progression of the G1-S phase in PDAC by regulating the expression of multiple cell cycle genes, including CDK2, CDK4 and CDK6, via p21 and p27 expression (Fig. 5). PDAC has a high frequency of inactivation of the CDKN2A (encoding the CDK4/6 inhibitor p16) (in > 80% of cases) and p53 (inducing the CDK2 inhibitor p21) (in 60-70% of cases) genes [3, 4], resulting in activated CDK2/4/6 and accelerated cancer progression. Recently, specific CDK4/6 inhibitors have been developed and used for advanced breast cancer [38]. Previous studies reported that CDK4/6 inhibitors also suppressed PDAC cell proliferation *in vitro* and *in vivo* [39], and some ongoing clinical trials are testing their efficacy in pancreatic cancer (ClinicalTrials.gov Identifier: NCT03065062, NCT04870034). However, it has also been reported that CDK4/6 inhibition monotherapy is not sufficient to inhibit the growth of PDAC cells [40]. In the current study, we indicated that selective blockade of RARγ signaling downregulated not only CDK4 and CDK6 but also dozens of other cell cycle genes, including CDK2, with increasing p21 and p27 expression, unlike CDK4/6 inhibitors. Another group also indicated that knockout of RARγ suppressed PDAC cell proliferation and downregulated cell cycle-related pathways, although cell cycle-related molecules were not addressed in detail [36]. Their results strongly support our data, which were obtained by knockdown experiments using two RARγ inhibitors. These findings suggest that selective blockade of RARγ signaling may be a more potent therapeutic strategy for PDAC than CDK4/6 inhibitors.

RARγ inhibition alone suppressed cell proliferation by downregulating many cell cycle genes but did not induce cell apoptosis. However, RARγ inhibition exerted synergistic tumor-suppressive effects with Gem by downregulating DNA repair genes. At present, DNA-damaging agents, such as Gem, 5-fluorouracil and oxaliplatin, or microtubule toxins, such as albumin-bound paclitaxel, are primarily used as chemotherapy for advanced PDAC [41]. However, no effective anticancer agent targets the cell cycle checkpoint in PDAC. Because these conventional chemotherapeutic agents target only dividing cells, there is concern that cell cycle-arresting agents, such as CDK4/6 inhibitors, impair the cytotoxic effects of these chemotherapeutic compounds. Previous studies have indeed documented that CDK4/6 inhibition antagonizes the effects of chemotherapy [42]. Recently, however, the combined effect of the two was reported to be enhanced by adjusting the order of administration: chemotherapeutic agent first, then CDK4/6 inhibitor [43]. Our current study demonstrated the enhancing effect of RARγ inhibition on Gem by adjusting the concentration to avoid completely arresting the cell cycle. Given that RARγ inhibition alone cannot induce cancer cell death but can inhibit DNA repair and that Gem and RARγ inhibition have different tumor-suppressive mechanisms, combining RARγ inhibition with DNA-damaging agents is rational. Our results suggest that the combination of DNA-damaging agents and RARγ inhibition is a promising new therapeutic strategy for PDAC in the future.

We clarified that PDAC expresses many esophagus-tissue-specific genes and that the increased expression of these genes was associated with a poor prognosis in PDAC patients. Furthermore, we showed that the expression of almost all esophagus-tissue-specific genes was positively correlated with that of RARγ and that RARγ signaling might regulate some esophagus-tissue-specific genes. Several studies have reported that in some types of cancer, including PDAC, the expression of squamous lineage markers, including esophagus-tissue-specific genes, correlated with a poor patient prognosis [8, 44, 45]. Recent studies have also suggested that a “basal-like/squamous type,” which expresses squamous lineage phenotypes, leads to PDAC progression and a poor prognosis [7]. However, the mechanism by which the acquisition of squamous lineage phenotypes leads to a poor patient prognosis is unclear. Our present results suggest that the activation of RARγ signaling might link squamous phenotypes with a poor prognosis in PDAC.

Our studies presented new insights into RARγ signaling in PDAC, but they are associated with several limitations: all of our experimental designs in this study focused only on cancer cell-autonomous functions associated with proliferation. This study did not evaluate other phenotypes, such as invasion and migration ability. In addition, the most significant limitation of this study is the lack of *in vivo* experiments. PDAC exhibits a high degree of heterogeneity in its molecular and histological features, which can affect the response to therapy. The samples used in this study were limited, and further research is needed to validate these findings using more samples and alternative models.

PDAC is a heterogeneous tumor characterized by a highly desmoplastic and immunosuppressive tumor microenvironment (TME) [46]. Cancer-associated fibroblasts (CAFs) play tumor-promoting and tumor-suppressing roles in the TME by activating some signaling pathways and producing an extracellular matrix [47]. RA signaling is also reported to involve the function of CAFs and the composition of immune cells in the TME [48, 49]. To elucidate whether RARγ signaling can genuinely be a therapeutic target for PDAC, we need to validate the tumor-suppressive effects of blocking RARγ signaling and clarify the association between RARγ signaling and the TME, including CAF function and immune cell composition, by analyzing not only cancer cells but also the tumor stroma using genetically engineered mouse models or patient-derived tumor xenograft models.

## Conclusions

This study clarified the function of RARγ signaling in PDAC progression and demonstrated the tumor-suppressive effect of selective blockade of RARγ signaling against PDAC. These results suggest that RARγ signaling might be a new therapeutic target for PDAC.

## Electronic supplementary material

Below is the link to the electronic supplementary material.

Additional file 1: **Table S1** Genetic information on the four major driver genes of PK-1, Panc-1 and PDAC organoids. **Table S2** Primer sequences used in RT‒PCR. **Table S3** Primary and secondary antibodies used in IHC. **Table S4** Primary and secondary antibodies used in Western blotting. **Table S5** The protein expression of esophagus-tissue-specific genes in PDAC. **Table S6** Top 50 pathways downregulated by RARγ inhibition.

Additional file 2: **S-Fig. 1** RARα and RARβ did not correlate with the patient prognosis of PDAC. **S-Fig. 2** Increased expression of esophagus-tissue-specific genes in PDAC correlated with a poor prognosis. **S-Fig. 3** Blockage of RARγ signaling suppressed PDAC cell proliferation. **S-Fig. 4** Blockage of RARγ signaling arrested the cell cycle in the G1 phase without causing cell death in PDAC cells. **S-Fig. 5** RARγ signaling did not cross-talk with the MAPK pathway. **S-Fig. 6** GSEA revealed that gene sets related to the cell cycle or DNA replication were downregulated by blocking RARγ signaling. **S-Fig. 7** Blockage of RARγ signaling upregulated the gene expression associated with the UPR in PDAC cells. **S-Fig. 8** Blockage of RARγ signaling induced increased expression of the endogenous CDK inhibitors p21 and p27 and decreased expression of p-CDK2/CDK2, CDK4 and CDK6. **S-Fig. 9** Blockage of RARγ signaling decreased the expression of some esophagus-tissue-specific genes. **S-Fig. 10** Blockage of RARγ signaling suppressed the proliferation of patient-derived PDAC organoids.

Additional file 3: The original images of blots for plots in Fig. 3d

## Data Availability

The RNA sequencing data of our in vitro experiments are available in GEO (accession number: GSE210112, URL: https://www.ncbi.nlm.nih.gov/geo). All the data generated during this study are included in this paper and its Supporting Information files.

## List of Abbreviations

ANOVA: Analysis of variance
ATRA: All-trans RA
CAF: Cancer-associated fibroblast
CDK: Cyclin-dependent kinase
DMSO: Dimethyl sulfoxide
FBS: Fetal bovine serum
FPKM: Fragments per kilobase of exon per million reads mapped
Gem: Gemcitabine
GEO: Gene Expression Omnibus
GSEA: Gene set enrichment analysis
GTEx: Genotype-Tissue Expression
HPA: Human Protein Atlas
IHC: Immunohistochemistry
OS: Overall survival
PanIN: Pancreatic intraepithelial neoplasia
PBS: Phosphate-buffered saline
PDAC: Pancreatic ductal adenocarcinoma
qPCR: Quantitative polymerase chain reaction
RA: Retinoic acid
RAR: Retinoic acid receptor
RARγ signaling: Retinoic acid signaling via retinoic acid receptor γ
RNA-seq: RNA sequencing
RT: Reverse transcription
SD: Standard deviation
siRNA: Small interfering RNA
TCGA: The Cancer Genome Atlas
TME: Tumor microenvironment
TPM: Transcripts per million
