# Supplementary material for: Blockage of retinoic acid signaling via RARγ suppressed the proliferation of pancreatic cancer cells by arresting the cell cycle progression of the G1-S phase
Source: Cancer Cell Int. 2023 May 17;23:94. doi: 10.1186/s12935-023-02928-4 (PMC10189913; doi:10.1186/s12935-023-02928-4)
Supplement: Supplementary file 1 — Additional file 1: Table S1 Genetic information on the four major driver genes of PK-1, Panc-1 and PDAC organoids. Table S2 Primer sequences used in RT‒PCR. Table S3 Primary and secondary antibodies used in IHC. Table S4 Primary and secondary antibodies used in Western blotting. Table S5 The protein expression of esophagus-tissue-specific genes in PDAC. Table S6 Top 50 pathways downregulated by RARγ inhibition. [file 12935_2023_2928_MOESM1_ESM.docx]

**Table S1** Genetic information on four major driver genes of PK-1, Panc-1 and PDAC organoids.

| **Cell name** | | **KRAS mutation** | **TP53 alteration** | **CDKN2A alteration** | **SMAD4 alteration** |
| --- | --- | --- | --- | --- | --- |
| PDAC cell lines | |  |  |  |  |
|  | PK-1 | Present (G12D) | Present (M237I) | Present (CNA) | Present (CNA) |
|  | Panc-1 | Present (G12D) | Present (R273H) | Present (CNA) | Absent |
| PDAC organoids | |  |  |  |  |
|  | KYK070 | Present (Q61H) | Present (R116Q) | Present (CNA) | Present (CNA) |
|  | KYK002 | Present (G12V) | Present (C9Y) | Present (CNA) | Present (CNA) |
|  | KYK023 | Present (G12D) | Present (Y73H) | Present (CNA) | Present (CNA) |
|  | KYK090 | Present (G12V) | Present (R141C) | Present (CNA) | Present (CNA) |
|  | KYK093 | Present (G12D) | Present (CNA) | Present (CNA) | Present (CNA) |

PDAC, pancreatic ductal adenocarcinoma; CNA, copy number alteration

**Table S2.** Primer sequences used in RT‒PCR

| **Primer name** | **Sequence (5' to 3')** | | **Size (bp)** |
| --- | --- | --- | --- |
| FABP5 | Fwd | TGA AGG AGC TAG GAG TGG GAA | 212 |
|  | Rev | TGC ACC ATC TGT AAA GTT GCA G |  |
| KRT13 | Fwd | CTG AAC AAG GAG GTG TCT ACC A | 162 |
|  | Rev | ATA GCG GCA CTC CGT CTC T |  |
| RARγ | Fwd | CTG TAT CAT CAA CAA GGT GAC CA | 182 |
|  | Rev | TGG TGA TGA GCT CTT CTA ACT GAG |  |
| ACTB | Fwd | CCT GGC ACC CAG CAC AAT | 70 |
|  | Rev | GCC GAT CCA CAC GGA GTA CT |  |

RT-PCR, reverse transcription polymerase chain reaction

**Table S3.** Primary and secondary antibodies used in IHC

| **Antibody** | **Dilution ratio** | **Catalog number** | **Source** |
| --- | --- | --- | --- |
| RARγ | 1:10 | sc-7387 | Santa Cruz Biotechnology |
| Ki67 | 1:250 | ab15580 | Abcam |
| AE1/AE3 | 1:20 | ab27988 | Abcam |
| Hoechst33342 | 1:10000 | H3570 | Invitrogen |
| Alexa Fluor 594-conjugated donkey anti-mouse IgG | 1:500 | A21203 | Invitrogen |
| Alexa Fluor 488-conjugated donkey anti-rabbit IgG | 1:500 | A21206 | Invitrogen |

IHC, immunohistochemistry

**Table S4.** Primary and secondary antibodies used in Western blotting

| **Antibody** | **Dilution ratio** | **Catalog number** | **Source** |
| --- | --- | --- | --- |
| CDK2 | 1:1000 | 2546 | Cell Signaling Technology |
| phospho-CDK2 | 1:1000 | 2561 | Cell Signaling Technology |
| CDK4 | 1:1000 | 12790 | Cell Signaling Technology |
| CDK6 | 1:1000 | 3136 | Cell Signaling Technology |
| p21 | 1:1000 | sc-6246 | Santa Cruz Biotechnology |
| p27 | 1:1000 | sc-1641 | Santa Cruz Biotechnology |
| p44/42 MAPK (Erk1/2) | 1:1000 | 4695 | Cell Signaling Technology |
| phospho-p44/42 MAPK (Erk1/2) | 1:1000 | 4370 | Cell Signaling Technology |
| RARγ | 1:200 | sc-7387 | Santa Cruz Biotechnology |
| FABP5 | 1:1000 | 12348-1-AP | Proteintech |
| KRT13 | 1:50000 | ab92551 | Abcam |
| β-actin | 1:3000 | A5441 | Sigma-Aldrich |
| HRP-conjugated anti-mouse IgG | 1:3000 | 7076 | Cell Signaling Technology |
| HRP-conjugated anti-rabbit IgG | 1:3000 | W4011 | Promega |

**Table S5.** The protein expression of esophagus tissue-specific genes in PDAC

| gene list | Protein expression |
| --- | --- |
| ECM1 | Present |
| KRT13 | Present |
| KRT4 | Present |
| KRT6A | Present |
| KRT6C | Present |
| KRT6B | Present |
| ERO1L | Present |
| FGFBP1 | Present |
| PADI1 | Present |
| DYNAP | Present |
| CRABP2 | Present |
| GJB2 | Present |
| IGFL1 | Absent |
| CLDN17 | Absent |
| CRNN | Absent |
| MUC21 | Absent |
| TMPRSS11E | Absent |
| KRTAP3-2 | Absent |
| TGM3 | Absent |
| KLK13 | Absent |
| A2ML1 | Absent |
| SERPINB13 | Absent |
| SERPINB3 | Absent |
| KRT78 | Absent |
| TGM1 | Absent |
| GBP6 | Absent |
| ADH7 | Absent |
| SCGB2A2 | Absent |
| MAL | n.a. |
| SPRR2B | n.a. |
| HMGN4 | n.a. |
| TMPRSS11B | n.a. |
| CAPN14 | n.a. |
| FAM83A | n.a. |
| UGT1A7 | n.a. |
| EPGN | n.a. |
| RAET1L | n.a. |
| MUC22 | n.a. |
| KRT32 | n.a. |
| KRT3 | n.a. |
| ZNF812 | n.a. |

n.a., not available; PDAC, pancreatic ductal adenocarcinoma

**Table S6.** Top 50 pathways downregulated by RARγ inhibition.

| WikiPathway Name | WikiPathway ID | p-value | Matched Entities | Pathway Entities |
| --- | --- | --- | --- | --- |
| Kinesins | WP1842 | 1.00E-12 | 11 | 43 |
| TP53 Regulates Transcription of Cell Cycle Genes | WP3804 | 1.00E-12 | 12 | 63 |
| Regulation of mitotic cell cycle | WP4109 | 1.00E-12 | 13 | 86 |
| Gastric Cancer Network 1 | WP2361 | 1.00E-12 | 10 | 29 |
| Retinoblastoma Gene in Cancer | WP2446 | 1.00E-12 | 40 | 90 |
| Mitotic G1-G1-S phases | WP1858 | 1.00E-12 | 55 | 169 |
| miRNA Regulation of DNA Damage Response | WP1530 | 1.00E-12 | 12 | 98 |
| ATM Signaling Pathway | WP2516 | 1.00E-12 | 9 | 41 |
| Mitotic G2-G2-M phases | WP1859 | 1.00E-12 | 22 | 190 |
| Mitotic Prophase | WP2654 | 1.00E-12 | 12 | 113 |
| Cell Cycle | WP179 | 1.00E-12 | 30 | 120 |
| G1 to S cell cycle control | WP45 | 1.00E-12 | 24 | 66 |
| Telomere Maintenance | WP1928 | 1.00E-12 | 13 | 64 |
| DNA IR-damage and cellular response via ATR | WP4016 | 1.00E-12 | 19 | 83 |
| DNA Damage Response | WP707 | 1.00E-12 | 11 | 68 |
| HDR through Homologous Recombination (HRR) or Single Strand Annealing (SSA) | WP3567 | 1.00E-12 | 17 | 114 |
| Base Excision Repair | WP4752 | 1.00E-12 | 8 | 31 |
| M-G1 Transition | WP2785 | 1.00E-12 | 21 | 85 |
| Mitotic Metaphase and Anaphase | WP2757 | 1.00E-12 | 32 | 184 |
| Mitotic Prometaphase | WP2652 | 1.00E-12 | 38 | 184 |
| Regulation of TP53 Activity through Phosphorylation | WP3838 | 1.00E-12 | 11 | 92 |
| Pyrimidine metabolism | WP4022 | 1.00E-12 | 13 | 99 |
| Regulation of DNA replication | WP1898 | 1.00E-12 | 14 | 76 |
| Cell Cycle Checkpoints | WP1775 | 1.00E-12 | 54 | 276 |
| Nucleosome assembly | WP1874 | 1.00E-12 | 12 | 54 |
| S Phase | WP2772 | 1.00E-12 | 11 | 101 |
| SUMOylation of DNA replication proteins | WP3805 | 1.00E-12 | 9 | 48 |
| DNA Replication Pre-Initiation | WP4461 | 1.00E-12 | 21 | 85 |
| Regulation of sister chromatid separation at the metaphase-anaphase transition | WP4240 | 1.00E-12 | 6 | 15 |
| DNA Replication | WP466 | 1.00E-12 | 22 | 42 |
| Synthesis of DNA | WP1925 | 6.64E-12 | 29 | 119 |
| RHO GTPases Activate Formins | WP3379 | 6.64E-12 | 26 | 123 |
| Gastric Cancer Network 2 | WP2363 | 4.91E-10 | 7 | 32 |
| Resolution of Abasic Sites (AP sites) | WP3345 | 1.91E-09 | 7 | 39 |
| DNA Mismatch Repair | WP531 | 3.16E-09 | 6 | 22 |
| Integrated Breast Cancer Pathway | WP1984 | 6.07E-09 | 11 | 170 |
| Transcriptional Regulation by E2F6 | WP4413 | 7.64E-09 | 7 | 44 |
| Integrated Cancer Pathway | WP1971 | 1.08E-08 | 7 | 49 |
| Mismatch Repair | WP3381 | 2.30E-08 | 5 | 15 |
| Nucleotide Metabolism | WP404 | 8.82E-08 | 5 | 19 |
| MHC class II antigen presentation | WP2679 | 1.45E-07 | 8 | 115 |
| Intra-Golgi and retrograde Golgi-to-ER traffic | WP3842 | 1.52E-07 | 10 | 179 |
| Nucleotide Excision Repair | WP4753 | 2.13E-07 | 6 | 43 |
| DNA Damage Bypass | WP1803 | 4.25E-07 | 6 | 49 |
| TP53 Regulates Transcription of DNA Repair Genes | WP3808 | 5.33E-07 | 7 | 87 |
| Ciliary landscape | WP4352 | 7.77E-07 | 10 | 214 |
| One Carbon Metabolism | WP241 | 8.58E-07 | 5 | 29 |
| miR-targeted genes in epithelium - TarBase | WP2002 | 1.17E-06 | 11 | 347 |
| Trans-sulfuration and one carbon metabolism | WP2525 | 1.22E-06 | 5 | 31 |
| DNA IR-Double Strand Breaks (DSBs) and cellular response via ATM | WP3959 | 1.23E-06 | 6 | 57 |
