# Supplementary material for: Blockage of retinoic acid signaling via RARγ suppressed the proliferation of pancreatic cancer cells by arresting the cell cycle progression of the G1-S phase
Source: Cancer Cell Int. 2023 May 17;23:94. doi: 10.1186/s12935-023-02928-4 (PMC10189913; doi:10.1186/s12935-023-02928-4)
Supplement: Supplementary file 2 — Additional file 2: S-Fig. 1 RARα and RARβ did not correlate with the patient prognosis of PDAC. S-Fig. 2 Increased expression of esophagus-tissue-specific genes in PDAC correlated with a poor prognosis. S-Fig. 3 Blockage of RARγ signaling suppressed PDAC cell proliferation. S-Fig. 4 Blockage of RARγ signaling arrested the cell cycle in the G1 phase without causing cell death in PDAC cells. S-Fig. 5 RARγ signaling did not cross-talk with the MAPK pathway. S-Fig. 6 GSEA revealed that gene sets related to the cell cycle or DNA replication were downregulated by blocking RARγ signaling. S-Fig. 7 Blockage of RARγ signaling upregulated the gene expression associated with the UPR in PDAC cells. S-Fig. 8 Blockage of RARγ signaling induced increased expression of the endogenous CDK inhibitors p21 and p27 and decreased expression of p-CDK2/CDK2, CDK4 and CDK6. S-Fig. 9 Blockage of RARγ signaling decreased the expression of some esophagus-tissue-specific genes. S-Fig. 10 Blockage of RARγ signaling suppressed the proliferation of patient-derived PDAC organoids. [file 12935_2023_2928_MOESM2_ESM.pdf]

Supplemental Figure 1

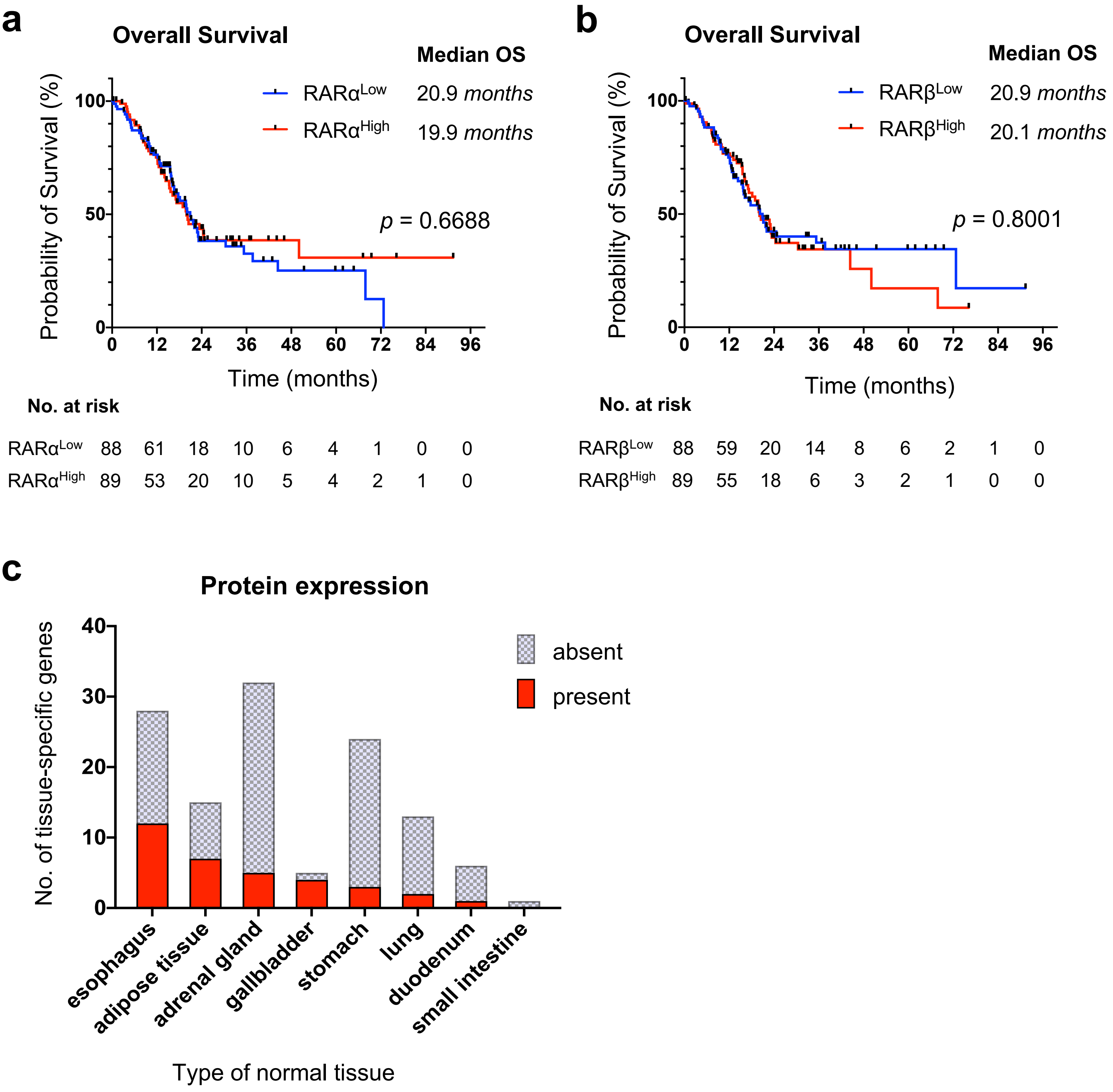

S-Fig. 1 RARα and RARβ did not correlate with the patient prognosis of PDAC.
a, b Pancreatic cancer patients were classified into $RAR\alpha^{High}$ or $RAR\alpha^{Low}$ and $RAR\beta^{High}$ or $RAR\beta^{Low}$ groups based on the transcript levels of RARα and RARβ, respectively, in TCGA-PAAD data, followed by estimating the patient OS using a Kaplan-Meier survival analysis. **c** The expression of proteins coded by tissue-specific genes in PDAC was assessed using the HPA. Genes with “High” or “Medium” protein expression in at least 1 of 8-12 PDAC sections were defined as having “present” protein expression in PDAC, and genes with “Low” or “not detected” protein expression in all PDAC sections were defined as having “absent” protein expression in PDAC. The bar charts represented the number of “present” and “absent” tissue-specific genes. *$p < 0.05$, **$p < 0.01$, ***$p < 0.001$, ****$p < 0.0001$; by log-rank test in **a, b**.

Supplemental Figure 2

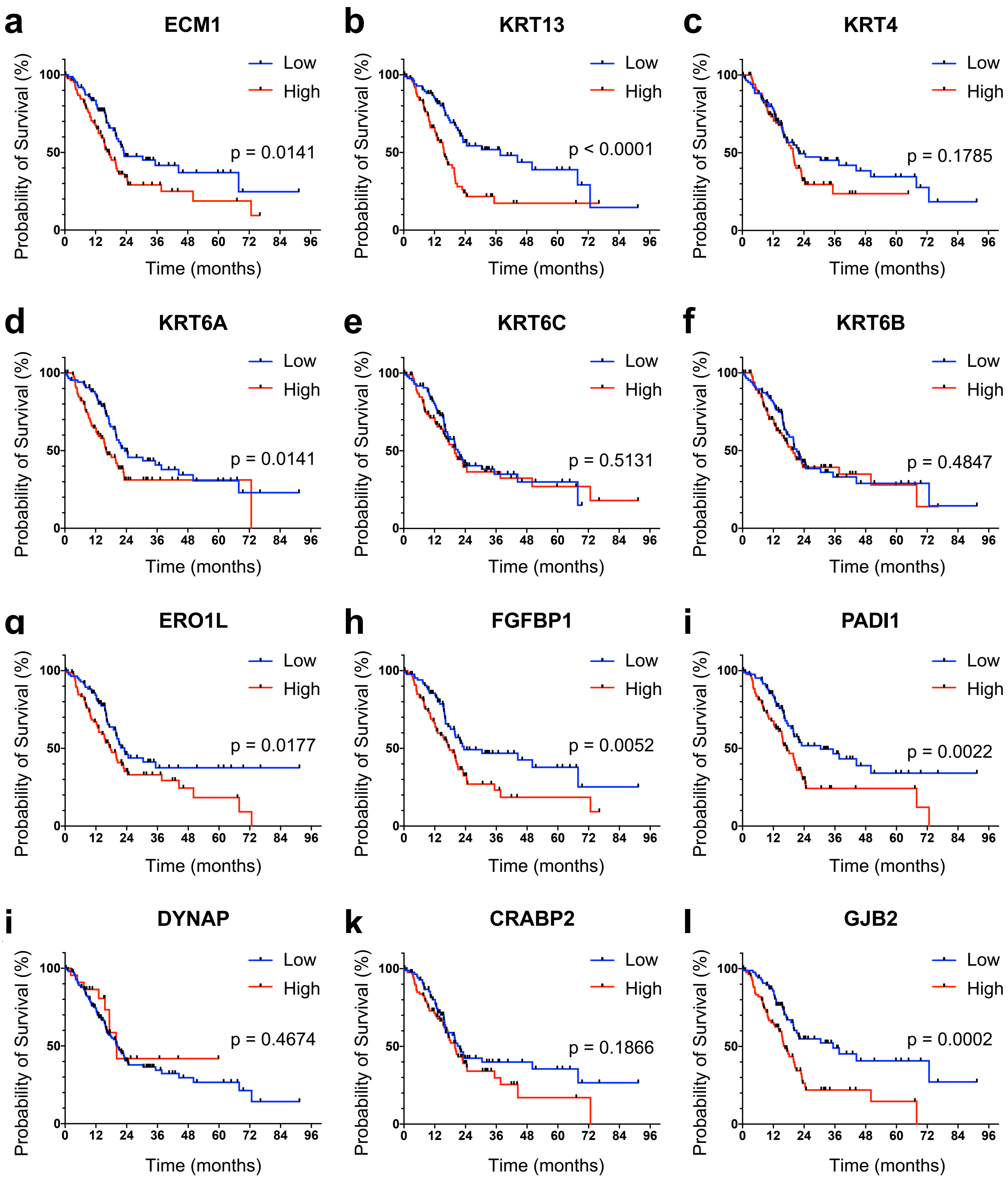

S-Fig. 2 Increased expression of esophagus tissue-specific genes in PDAC correlated with a poor prognosis.

a-l Pancreatic cancer patients were classified into high- or low-expression groups based on the transcript levels of each esophagus-specific gene in TCGA-PAAD data, and then the patient OS was compared using a Kaplan-Meier survival analysis and log-rank test.

Supplemental Figure 3

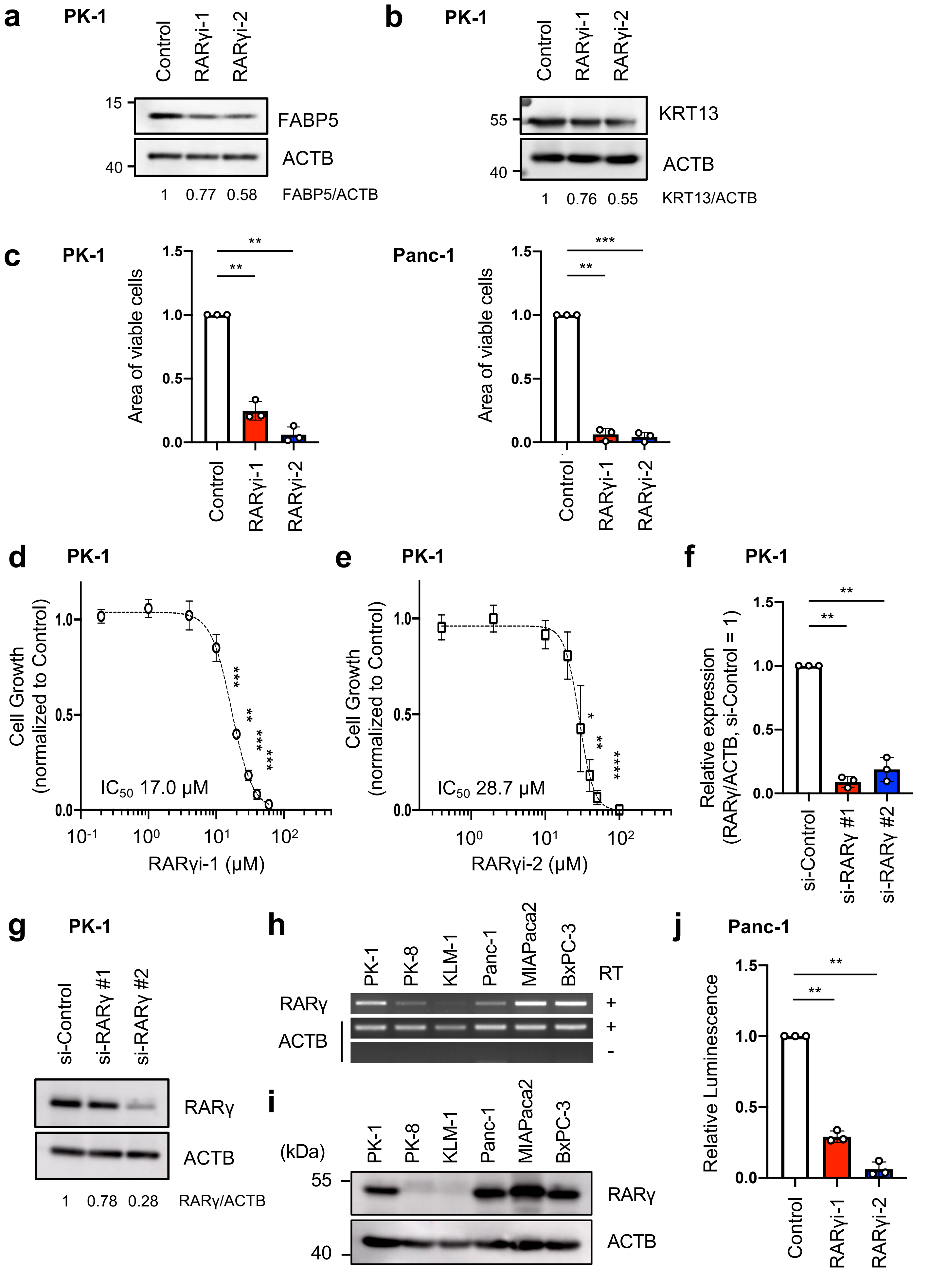

S-Fig. 3 Blockage of RARγ signaling suppressed PDAC cell proliferation.
a, b The protein expressions of FABP5 (a) and KRT13 (b) were assessed using Western blotting 24 h after RARγ inhibition in PK-1 cells. **c** The area of viable cells was assessed and quantified by crystal violet staining and the ImageJ software program on day 3 after RARγ inhibition in PK-1 and Panc-1 cells. The area of viable cells surrounded by the red line (in Fig. 2d) was quantified, and that in the control was normalized to 1. **d, e** The number of viable cells was assessed on day 3 after RARγi-1 or RARγi-2 treatment by an ATP assay in PK-1, and then the luminescence was normalized to control (0 μM RARγi-1 or RARγi-2). **f** The mRNA expression of RARγ was assessed using qPCR on day 2 after RARγ inhibition in PK-1 cells. **g** The protein expression of RARγ was assessed using Western blotting on day 4 after RARγ inhibition in PK-1 cells. **h, i** The mRNA (g) and protein (h) expression of RARγ was assessed using semiquantitative PCR and Western blotting, respectively, in various PDAC cell lines. **j** The number of viable cells was assessed on day 3 after RARγ inhibition treatment by an ATP assay in Panc-1 cells. Error bars in **c-e, i**, mean ± SD of three independent experiments; *p < 0.05, **p < 0.01, ***p < 0.001, ****p < 0.0001; n.s., not significant; by a one-way ANOVA with Dunnett's test (compared to control) in **c-f, j**.

Supplemental Figure 4

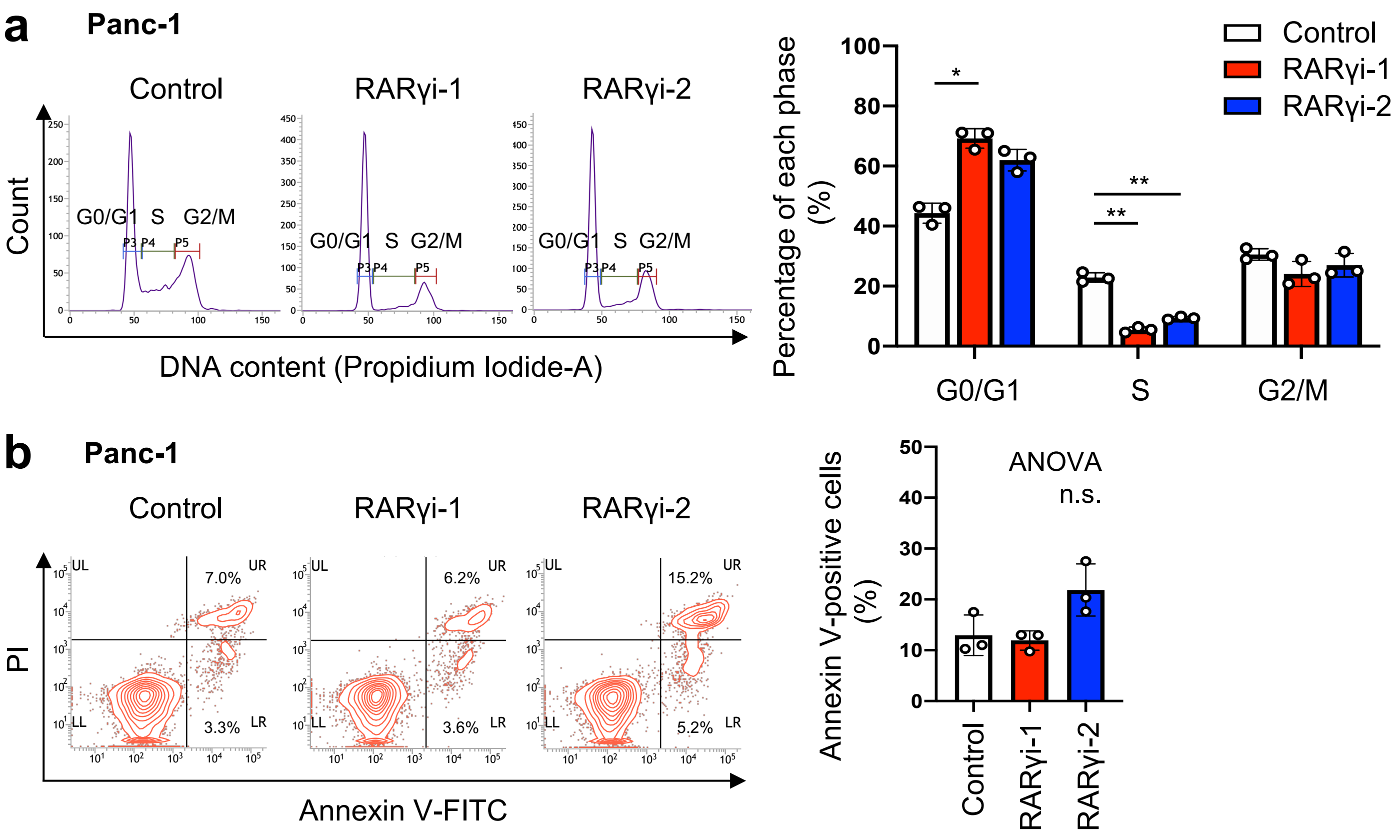

S-Fig. 4 Blockage of RARγ signaling arrested the cell cycle in the G1 phase without causing cell death in PDAC cells.

a Left, representative images of a cell cycle analysis 24 h after RARγ inhibition in Panc-1 cells. Right, the percentages of cells in each phase of the cell cycle 24 h after RARγ inhibition are shown from three independent experiments. **b** Left, representative images of a flow cytometry analysis 24 h after RARγ inhibition in Panc-1 cells. Right, the percentages of annexin V-positive Panc-1 cells 24 h after RARγ inhibition are shown from three independent experiments. Error bars in **a, b**, mean ± SD of three independent experiments; *$p < 0.05$, **$p < 0.01$, ***$p < 0.001$, ****$p < 0.0001$; n.s., not significant; by a one-way ANOVA with Dunnett's test (compared to control) in **a**, or a one-way ANOVA in **b**.

Supplemental Figure 5

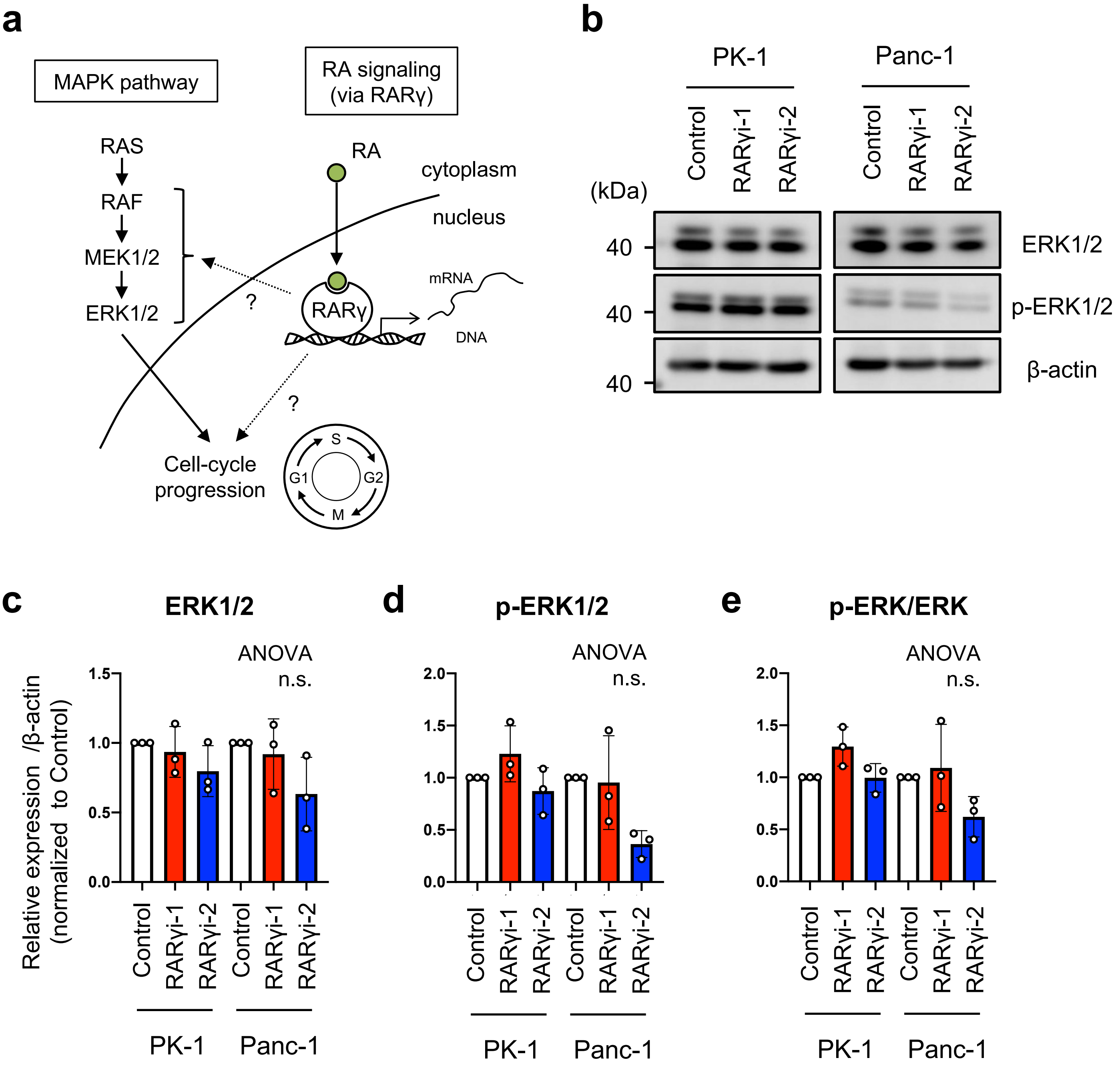

S-Fig. 5 RARγ signaling did not cross-talk with the MAPK pathway.
a Schematic illustration of whether or not RARγ signaling cross-talked with the MAPK pathway. **b** Representative images, the protein expression and phosphorylation of ERK1/2 were assessed using Western blotting 24 h after RARγ inhibition in PK-1 and Panc-1 cells. **c-e** The protein expression and phosphorylation of ERK1/2 24 h after RARγ inhibition are quantified using the ImageJ software program and shown from three independent experiments. Error bars in **c-e**, mean ± SD of three independent experiments; n.s., not significant; by a one-way ANOVA in **c-e**.

Supplemental Figure 6

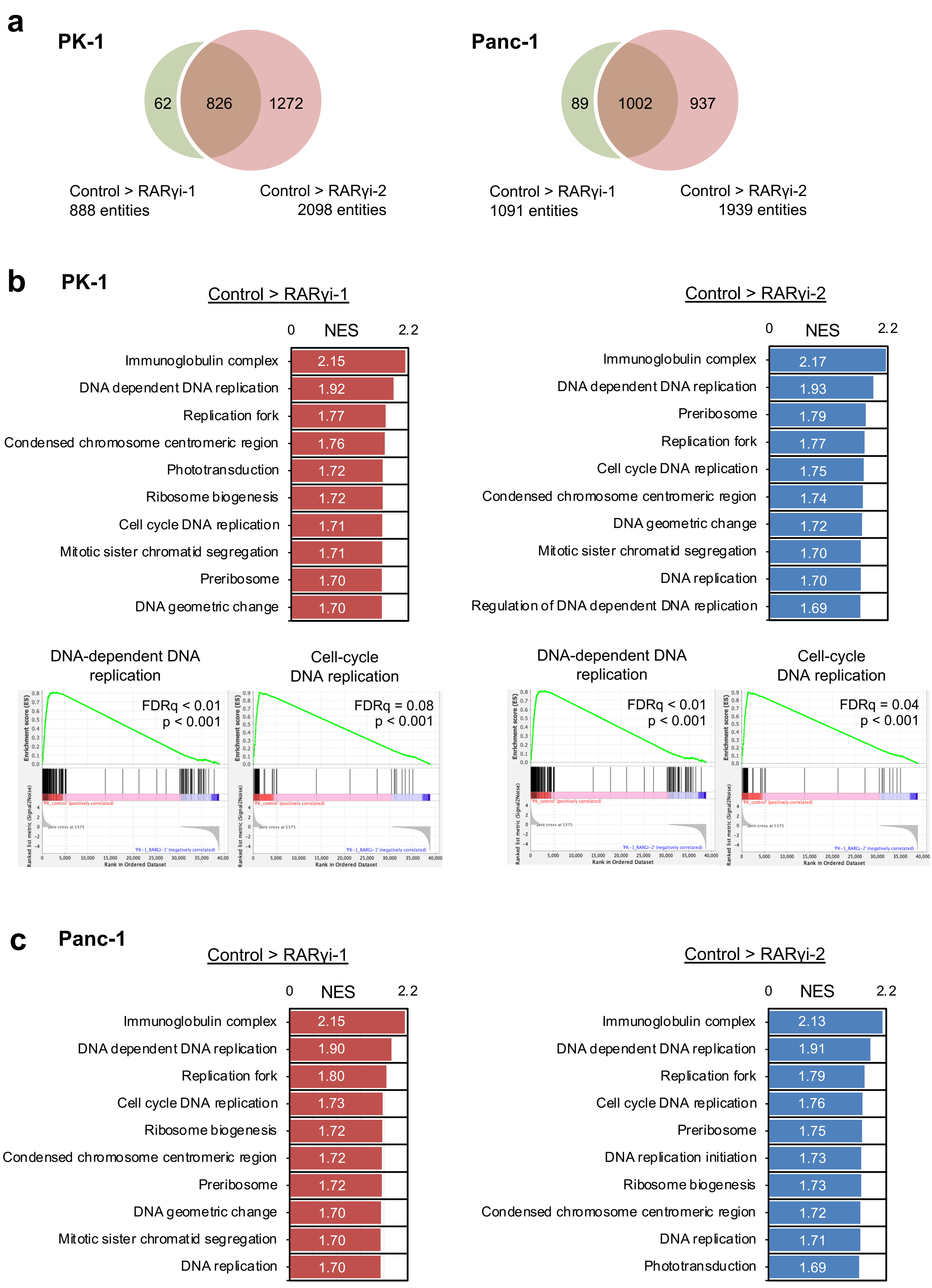

S-Fig. 6 GSEA revealed that gene sets related to the cell cycle or DNA replication were downregulated by blocking RARγ signaling.

a Venn diagrams show the entities whose expression was down-regulated by RARγi-1 or RARγi-2 treatment in PK-1 (left) and Panc-1 (right) cells. **b, c** A GSEA was performed using RNA sequencing data from PK-1 (b) and Panc-1 (c) cells treated with RARγi-1 or RARγi-2. The red and blue bars indicate the top 10 gene sets downregulated by RARγi-1 and RARγi-2 treatment, respectively. **b** Bottom, Representative GSEA plots. NES, normalized enrichment score; FDR, false discovery rate.

Supplemental Figure 7

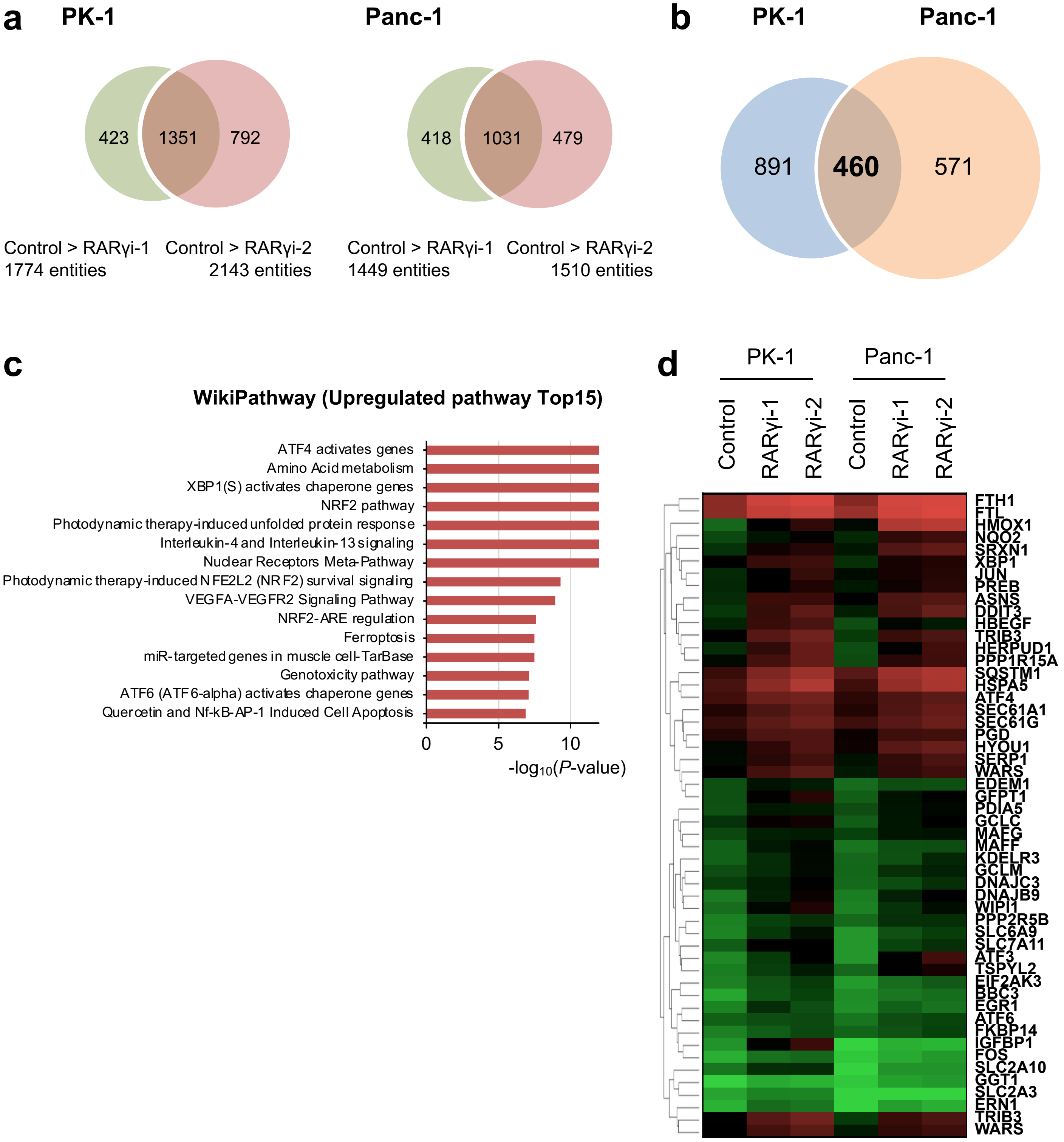

S-Fig. 7 Blockage of RARγ signaling upregulated the gene expression associated with the UPR in PDAC cells.

a Venn diagrams show the entities whose expression was up-regulated by RARγi-1 or RARγi-2 treatment in PK-1 (left) and Panc-1 (right) cells. **b** Venn diagrams show the entities whose expression was up-regulated by RARγ inhibition in PK-1 and Panc-1. **c** The red bars indicate the top 15 pathways up-regulated by RARγ inhibition. **d** The heatmap of the RNA sequencing experiment shows the expression of entities selected from ATF4 activates genes (WP2753), XBP1(S) activates chaperone genes (WP3472), NRF2 pathway (WP2884), Photodynamic therapy-induced unfolded protein response (WP3613), and Photodynamic therapy-induced NFE2L2 (NRF2) survival signaling (WP3612) in **c**.

Supplemental Figure 8

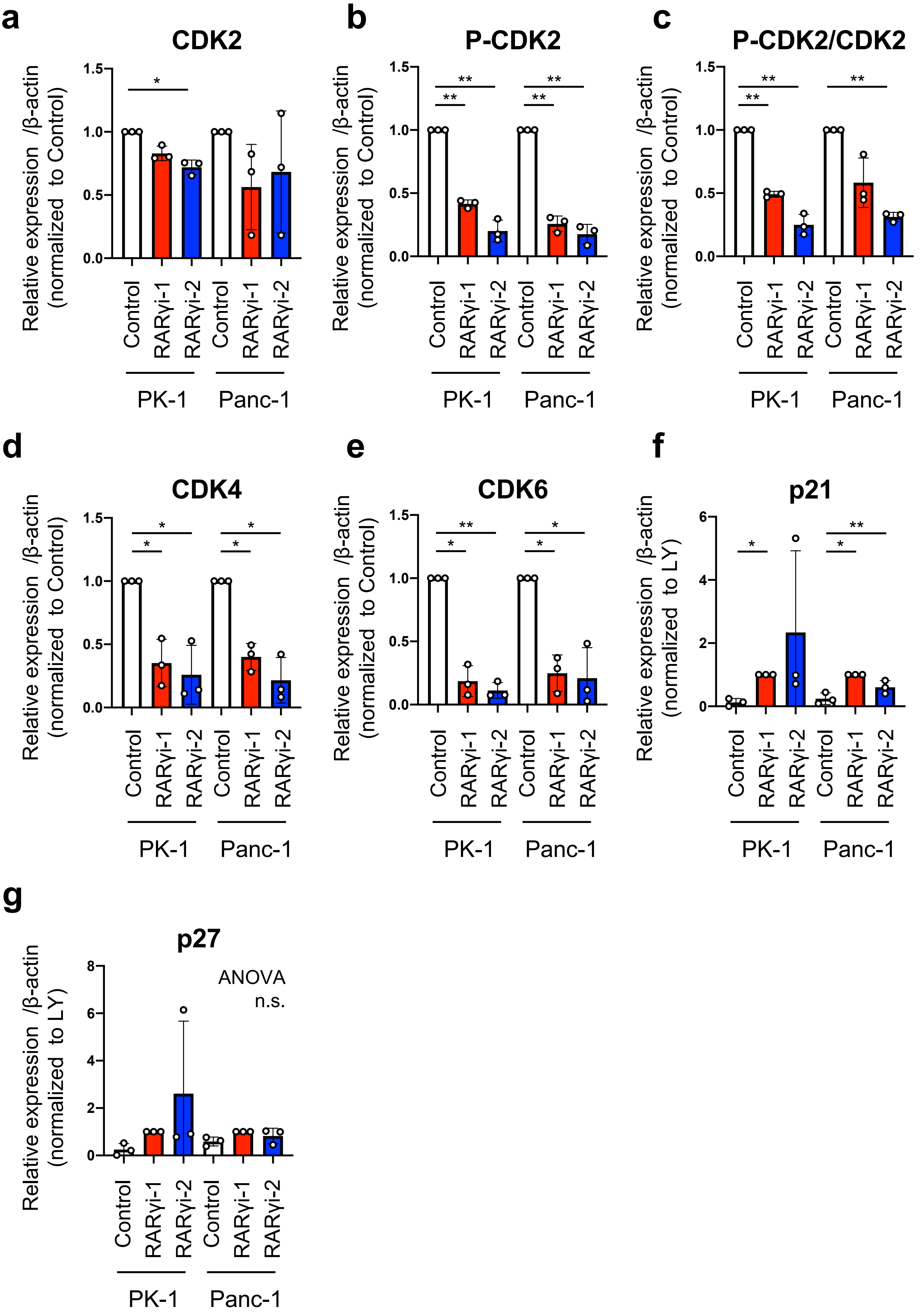

S-Fig. 8 Blockage of RARγ signaling induced increased expression of the endogenous CDK inhibitors p21 and p27 and decreased expression of p-CDK2/CDK2, CDK4 and CDK6.

a-f The protein expression of CDK2, p-CDK2, CDK4, CDK6, p21 and p27 24 h after RARγ inhibition are quantified using the ImageJ software program and shown from three independent experiments. Error bars in **a-g**, mean ± SD of three independent experiments; $*p < 0.05$, $**p < 0.01$, $***p < 0.001$, $****p < 0.0001$; n.s., not significant; by a one-way ANOVA with Dunnett's test (compared to control) in each cell line in **a-g**.

Supplemental Figure 9

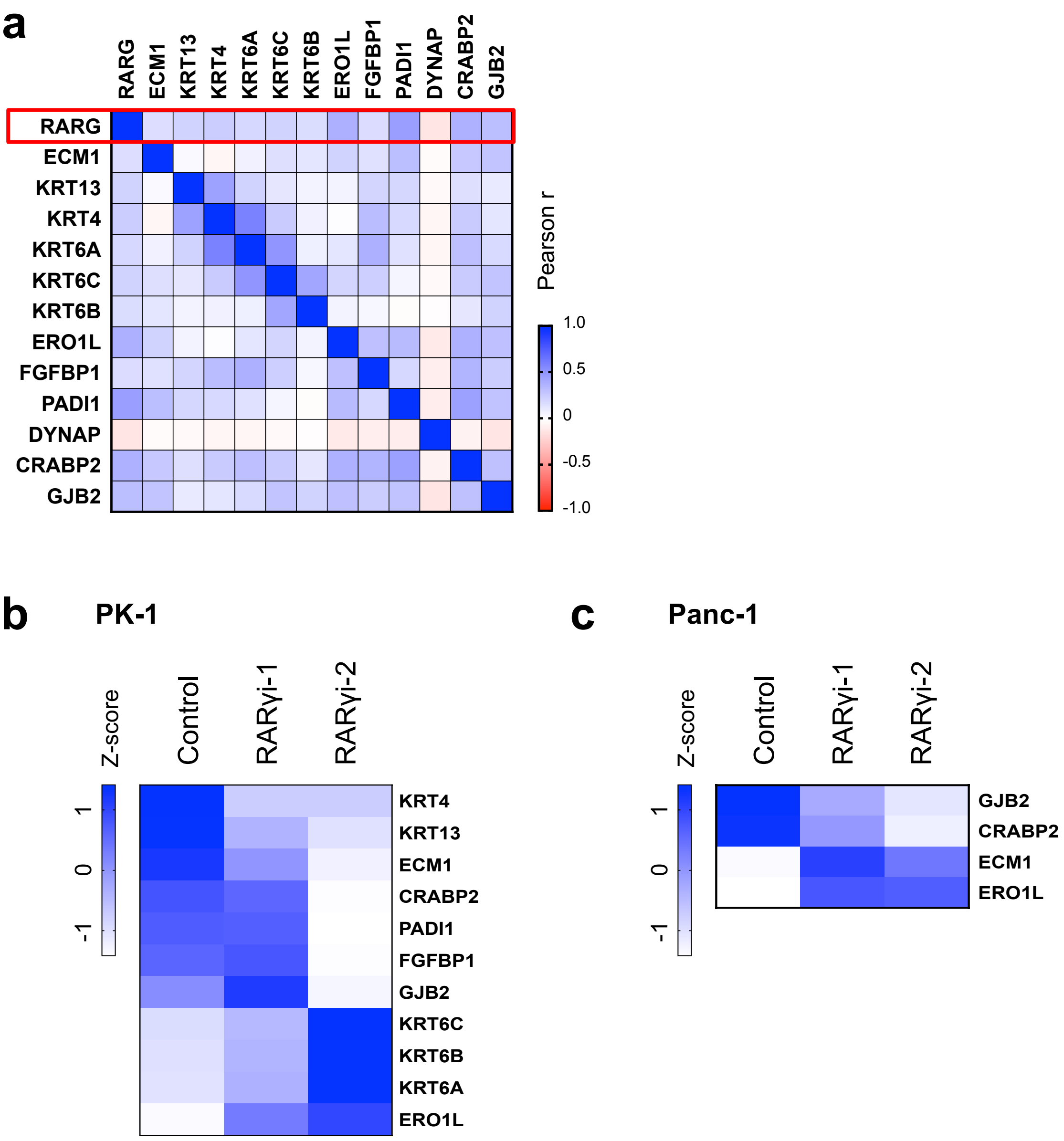

S-Fig. 9 Blockage of RARγ signaling decreased the expression of only a few esophagus-tissue-specific genes.

a The heatmap indicates the correlations between RARγ and esophagus tissue-specific genes with confirmed protein expression in PDAC (listed by Table S5) in TCGA-PAAD cases. **b, c** Heatmap from RNA sequencing data showed the expression of esophagus-tissue-specific genes with confirmed protein expression in PDAC (listed by Table S5) in PK-1 (b) and Panc-1 (c). The heatmap excluded the genes with TPM values < 1 according to RNA sequencing in all samples.

Supplemental Figure 10

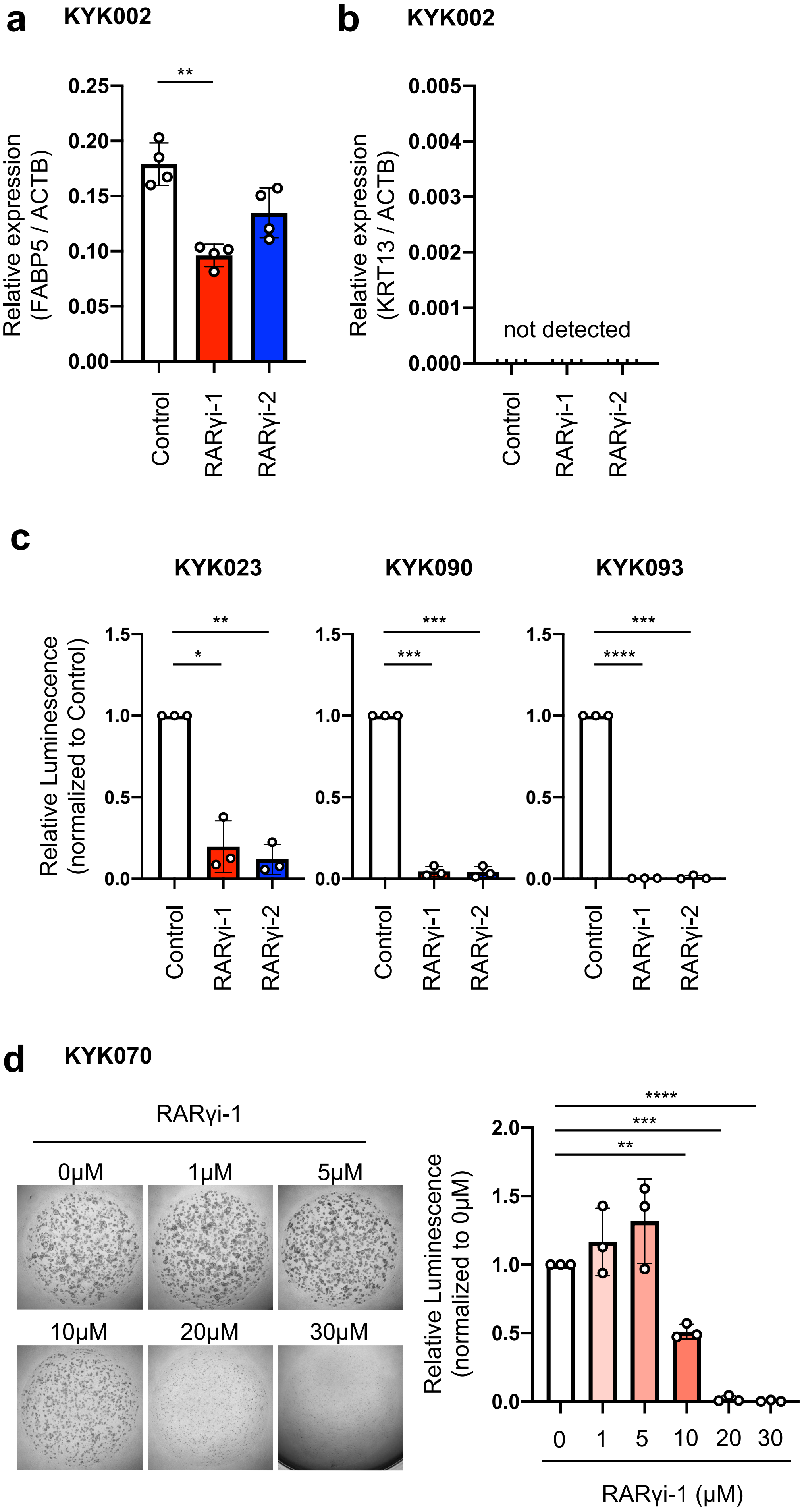

S-Fig. 10 Blockage of RARγ signaling suppressed the proliferation of patient-derived PDAC organoids.
a, b The transcript levels of FABP5 (a) and KRT13 (b) were measured using qPCR 24 h after RARγ inhibition in KYK002 organoids. **c** The number of viable cells was assessed by an ATP assay after RARγ inhibition for 10 days in KYK023, KYK090 and KYK093 organoids. **d** Left, representative images after the treatment with various concentrations of RARγi-1 for 10 days. Right, KYK070 organoids were treated with various concentrations of RARγi-1 for 10 days. The number of viable cells was assessed by an ATP assay, and then the luminescence was normalized to control (0 μM RARγi-1). Error bars in **a-c,** mean ± SD of three independent experiments; *p < 0.05, **p < 0.01, ***p < 0.001, ****p < 0.0001; by a one-way ANOVA with Dunnett's test (compared to control) in **a, c, d**.
