# Supplementary figures and images for: Blockage of retinoic acid signaling via RARγ suppressed the proliferation of pancreatic cancer cells by arresting the cell cycle progression of the G1-S phase
Source: Cancer Cell Int. 2023 May 17;23:94. doi: 10.1186/s12935-023-02928-4 (PMC10189913; doi:10.1186/s12935-023-02928-4)

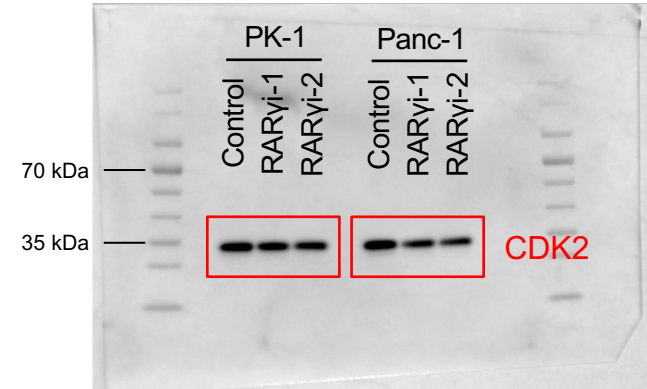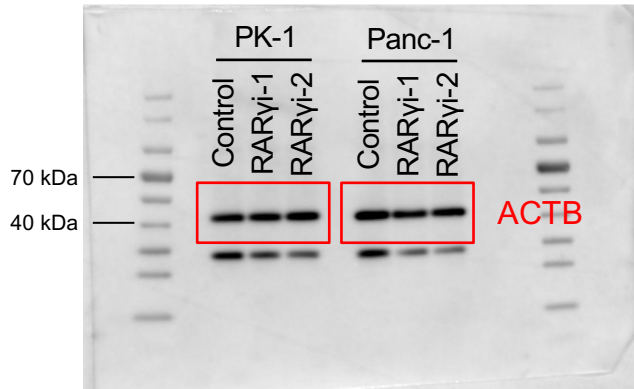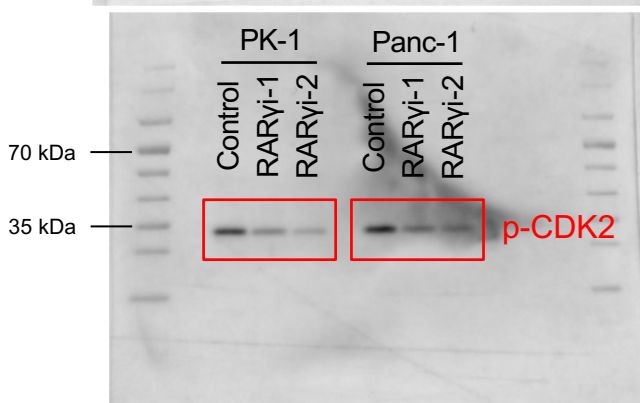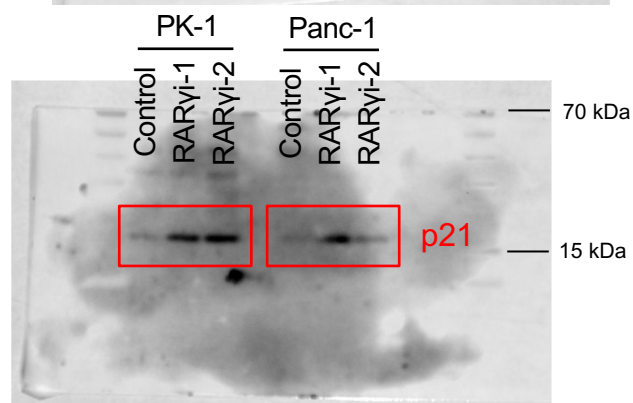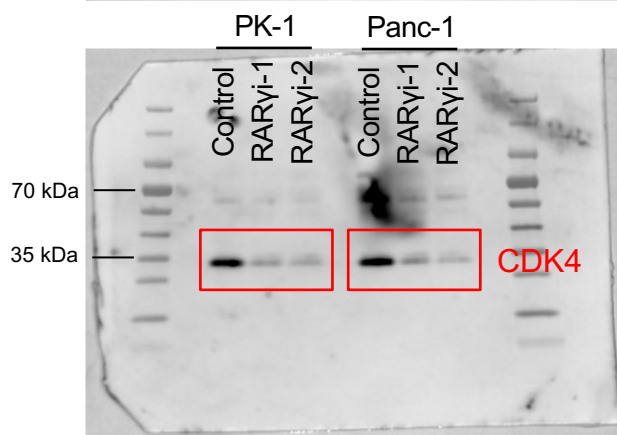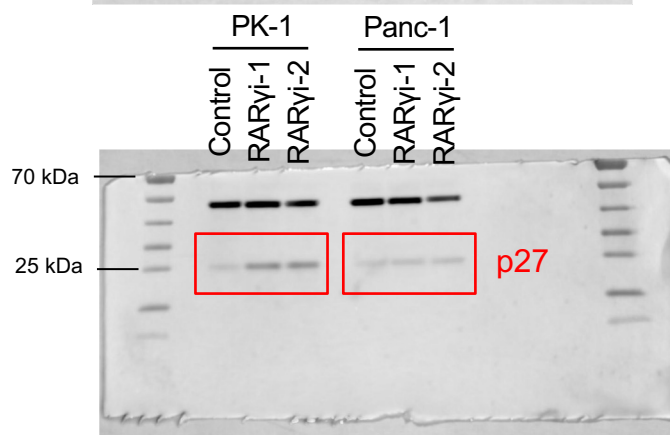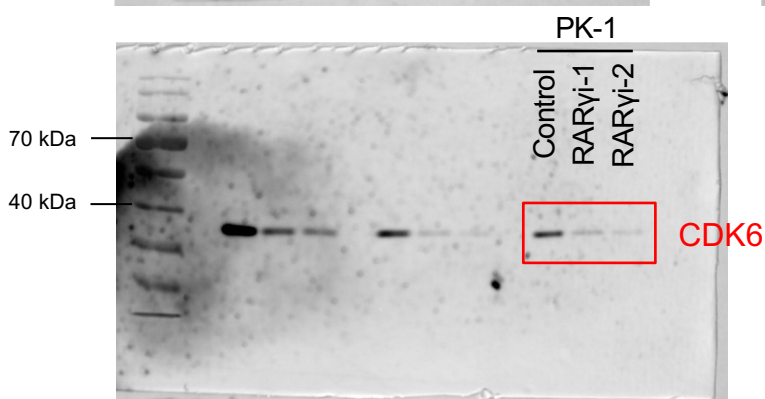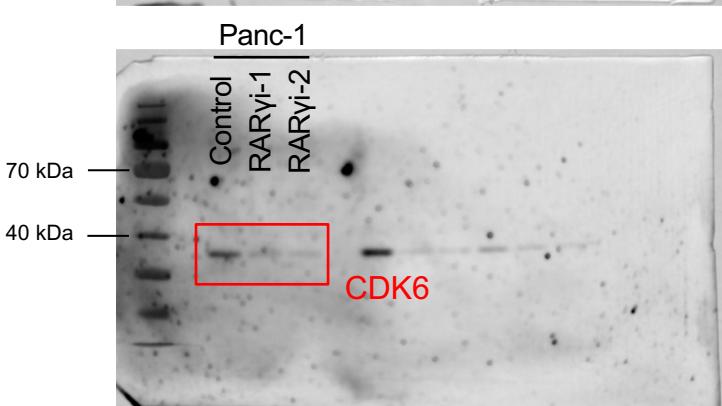

Supplement: Supplementary file 3 — Additional file 3: The original images of blots for plots in Fig. 3d [file 12935_2023_2928_MOESM3_ESM.pdf]
